# Photoluminescent Metal–Organic Frameworks for Gas Sensing

**DOI:** 10.1002/advs.201500434

**Published:** 2016-03-15

**Authors:** Rui‐Biao Lin, Si‐Yang Liu, Jia‐Wen Ye, Xu‐Yu Li, Jie‐Peng Zhang

**Affiliations:** ^1^ MOE Key Laboratory of Bioinorganic and Synthetic ChemistrySchool of Chemistry and Chemical EngineeringSun Yat‐Sen UniversityGuangzhou510275P.R. China

**Keywords:** gas, luminescence, porous coordination polymer, sensor, vapor

## Abstract

Luminescence of porous coordination polymers (PCPs) or metal–organic frameworks (MOFs) is sensitive to the type and concentration of chemical species in the surrounding environment, because these materials combine the advantages of the highly regular porous structures and various luminescence mechanisms, as well as diversified host‐guest interactions. In the past few years, luminescent MOFs have attracted more and more attention for chemical sensing of gas‐phase analytes, including common gases and vapors of solids/liquids. While liquid‐phase and gas‐phase luminescence sensing by MOFs share similar mechanisms such as host‐guest electron and/or energy transfer, exiplex formation, and guest‐perturbing of excited‐state energy level and radiation pathways, via various types of host‐guest interactions, gas‐phase sensing has its unique advantages and challenges, such as easy utilization of encapsulated guest luminophores and difficulty for accurate measurement of the intensity change. This review summarizes recent progresses by using luminescent MOFs as reusable sensing materials for detection of gases and vapors of solids/liquids especially for O_2_, highlighting various strategies for improving the sensitivity, selectivity, stability, and accuracy, reducing the materials cost, and developing related devices.

## 1 Introduction

The most common and typical methods that have been deve­loped for the purpose of chemical analyses are mainly based on chromatography, optical absorption spectroscopy, and electrochemistry. However, most of these techniques require complexed instruments and time‐consuming pretreatment steps, restricting their applications for in‐field and real‐time detection. Compared with other transduction techniques, luminescence is the preferred signal for sensing, because of its visibility to the naked eye, well‐developed technique, extremely low detection limits, and also simple sample preparation. There are great needs for sensitive and selective detection of gaseous analytes from laboratory to industry.^1^ Gas sensors have a wide range of applications including managing industrial process, controlling food quality, medical diagnostics, occupational safety, detecting chemical threat, and monitoring environment.^2^, ^3^, ^4^ While commercialized sensors relying on organic‐polymeric or inorganic‐semiconductor materials have been well developed, there are always new requirements and challenges, as well as great space for their development.

Porous coordination polymers (PCPs) or metal–organic frameworks (MOFs) are unparalleled for their diversified, desi­gnable and tailorable structures as well as unique chemical and physical properties.^5^, ^6^, ^7^, ^8^, ^9^, ^10^ Over the past two decades, MOFs have attracted extensive interest for their great potential in many applications such as gas separation/storage,^11^, ^12^, ^13^, ^14^, ^15^, ^16^, ^17^ catalysis,^18^, ^19^, ^20^ drug delivery,^21^ and luminescence sensing.^2^, ^3^, ^4^, ^22^, ^23^ As for conventional metal complexes, photoluminescence properties of MOFs can be finely tuned by rational selection of metal ions and design of organic ligands. Since the metal ions and ligands in MOFs are arranged in highly ordered arrays with controllable spatial distribution,^24^, ^25^ self‐quenching between adjacent luminophores can be reduced or avoided, giving enhanced luminescence intensity. In addition, luminescence can also be generated from guest chromophore or charge transfer between the host framework and guest molecules. The diversified luminescence mechanisms make MOFs a unique platform for various luminescence applications.^2^, ^3^, ^4^, ^26^, ^27^, ^28^, ^29^

Similar with common luminescent materials, the luminescence of MOFs can be influenced by many environmental factors,^3^, ^4^ including physical stimulus and chemical stimulus (ions,^30^, ^31^, ^32^, ^33^ solvents,^34^, ^35^, ^36^ vapors,^37^ pH,^38^, ^39^ gases,^1^ and so on^40^, ^41^, ^42^, ^43^, ^44^, ^45^). However, unlike other luminescent materials, the intrinsic porosity, large surface area, and strong adsorption affinity of MOFs can effectively enrich guest species. The guest analytes can interact with the host framework through not only weak van der Waals interactions but also hydrogen bonding, π–π stacking and even coordination bonds, which can effectively alter the luminescence emission intensity and/or color of the host by changing its excited‐state energy, non‐radiation pathway and efficiency, as well as the ground‐ and excited‐state host structure. Therefore, luminescence of MOFs can be very sensitive to slight changes of the chemical environment. Generally, the physically adsorbed guest species can be desorbed easily, which enables luminescent MOFs for non‐destructive, continuous and recyclable sensing. Since MOFs can be rationally designed with specific functional groups, appropriate pore sizes and tunable electronic structures, their intrinsic luminescence properties and host‐guest interactions can be rationally tuned to fit specific chemical sensing applications. The mole­cular sieving effect and/or structural transformations of some MOFs which selectively adsorb some guest over others could be used to achieve high sensing selectivity, which is hardly available for conventional luminescence materials.

The guest species that can be sensed by luminescent MOFs exist in the liquid and/or gas phases. Because liquids are closely related to the surrounding environment and human body, many materials (including MOFs) have been developed for liquid‐phase luminescence sensing of ionic and molecular species.^46^, ^47^, ^48^, ^49^, ^50^, ^51^, ^52^, ^53^ In the liquid environment, the types and concentrations of the analytes can be easily controlled for quantitative detection (calibration). To avoid undesired bias/fluctuation of the luminescence signal, MOF materials are usually prepared as fined powders to achieve stable dispersion in the target liquid (similar with the dissolved molecular luminescence probes). In comparison, the luminescent intensity of bulk MOF powder sample is related to not only the type and concentration of the analyte but also many other factors such as the particle size, surface roughness, and placement of the sample, which are highly dependent on the sample preparation method/history and gas flow/pressure, being unfavorable for quantitative detection. Further, many gaseous species interact weakly with MOFs and the partial pressure of gaseous analyte is usually difficult to control (especially for those with low saturation pressures), as compared with other ionic and/or heavier species dissolved in liquids. Therefore, when luminescent MOFs are used for gas‐phase sensing, gas analytes are generally presented as saturated vapors, and only the emission colors (frequencies/wavelengths) are compared in detail. Nevertheless, the fast increasing number of MOF materials and their thin‐film fabrication techniques,^54^, ^55^, ^56^ coupled with the ever growing demand for accurate detection of the chemical information of air and many gaseous environments,^1^, ^57^, ^58^, ^59^, ^60^, ^61^ have stimulated more and more studies using luminescent MOFs as sensing materials for the gas environment.^46^, ^62^, ^63^, ^64^

In the past few years, the range of detectable gases and sensing performances of luminescent MOFs have rapidly developed. This review aims to provide a timely overview of the current state of luminescent MOFs as reusable sensing materials for non‐destructive detection of gas‐phase analytes, with representative examples mainly categorized based on their sensing mechanisms, i.e., the roles of framework flexibility, coordinative guests, nitro‐containing and aromatic compounds, and radical gases.

## 2 Flexibility of MOFs

Framework flexibility is a unique advantage of MOFs,^65^, ^66^ which is not only interesting for the notable structural variations under external stimuli, but also important for their usefulness in a wide range of applications such as storage,^67^, ^68^ separation,^69^, ^70^, ^71^, ^72^, ^73^, ^74^ and sensing.^75^, ^76^, ^77^, ^78^, ^79^ Actually, even trivial (unnoticeable or hardly detectable) framework flexibility has fundamental importance for luminescence, because the transient motion (vibration, torsion, etc.) of organic luminophores at the excited state is the main cause of non‐radiative relaxation of the excited‐state energy.

**Figure 1:**
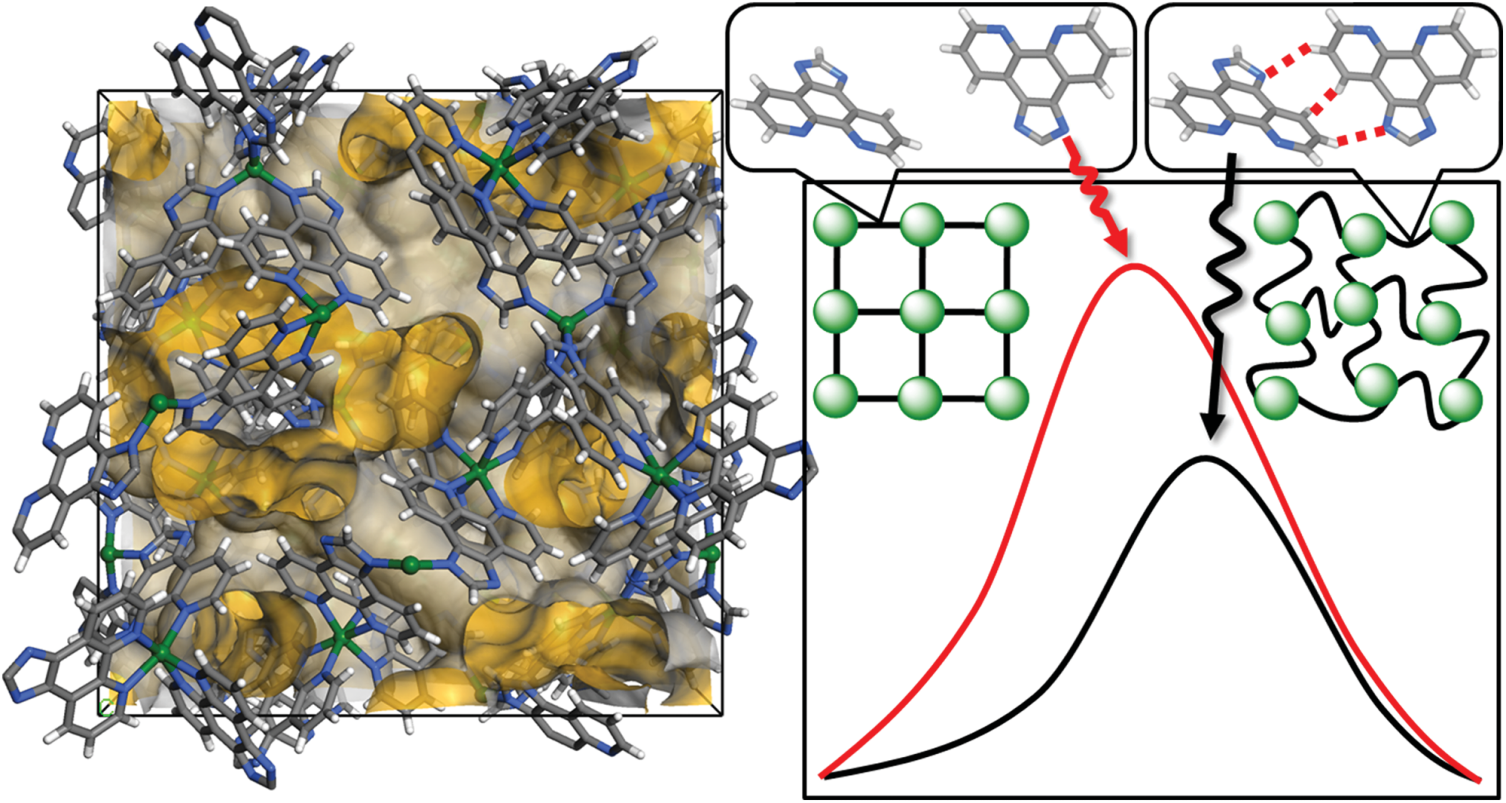
Structure of MAF‐34 and illustration of its fluorescence mechanism controlled by its crystalline/amorphous degree which determines intermolecular interactions between adjacent ip^−^ ligands.

Obviously, large structural variations of MOFs at the ground state can have great influence on the luminescence properties.^80^ For example, we showed that the flexibility of a porous metal azolate framework [Zn_7_(ip)_12_](OH)_2_ (MAF‐34, Hip = 1*H*‐Imidazo[4,5‐*f*][1,10]phenanthroline) is fundamental for its drastic fluorescence responses toward a variety of solvent vapors and CO_2_ (**Figure** **1**).^81^ MAF‐34 is an ultramicroporous C_3_N_4_‐type (**ctn**) network interconnected by [Ru(22bpy)_3_]^2+^ (22bpy = 2,2′‐bipyridine) like {Zn(ip)_3_}^–^ and tetradedral zinc‐imidazolate {Zn(ip)_4_}^2–^ fragments. Because the three‐coordinated ip^–^ ligands cannot fulfill the trigonal‐planar geometry for the 3‐connected node in the **ctn** net, {Zn(ip)_3_}^–^ adopts an obviously distorted octahedral coordination geometry which leads to significant framework tension. Therefore, MAF‐34 exhibited reversible crystal‐to‐amorphous structural transformations during guest adsorption, desorption, and exchange. The ip^−^ ligands in the as‐synthesized crystalline framework are well separated by the Zn(II) ions to avoid the typical face‐to‐face π–π interactions of this type of large planar aromatic molecules, although they are still close to each other with notable edge‐to‐edge interactions. After removal of the guest solvent molecules, the adjacent ip^−^ ligands become closer when the framework is distorted in the quasi‐amorphous phase. Therefore, the as‐synthesized MOF exhibited strong cyan fluorescence maximum at 487 nm, and the guest‐free phase exhibited orange emission centered at 554 nm. For comparison, the diluted solution of Hip shows blue fluorescence at 440 nm and crystalline Hip has no observable luminescence due to the serious π–π interactions. Guest‐free MAF‐34 showed reversible luminescence responses toward saturated MeOH, EtOH, H_2_O, benzene and nitrobenzene vapors. While electrons transfer from host framework to nitrobenzene accounted for the fluorescence quenching, the luminescence responses for other vapors should arise from the guest‐dependent network distortion, where the more crystalline ones (MeOH and EtOH) showed shorter emission wavelengths, and the more amorphous ones (H_2_O and benzene) showed longer emission wavelengths, because of the different extents of excimer formation controlled by the ligand‐ligand distances. Interestingly, when CO_2_ pressure changed from 4 to 90 Pa, desolvated MAF‐34 showed gradually blue‐shifted (from 554 to 540 nm) and enhanced fluorescence (ca. 60%). Microcrystalline thin film of MAF‐34 can be directly grown on zinc slice by using the substrate instead of Zn(II) salt as a reactant. To quantitative monitor the intensity change, in situ de‐gas and introduction of CO_2_ were conducted in a sealed chamber equipped with quartz windows and a three‐way valve which connects the chamber to a vacuum pump and a CO_2_ cylinder, and all luminescent response processes could be repeated for at least three cycles. The strong CO_2_ adsorption affinity of the uncoordinated imidazolate N donors and hydroxide anions should have played important roles, which enable adsorption of large amounts of CO_2_ to induce the amorphous‐to‐crystal structural transformation.

**Figure 2:**
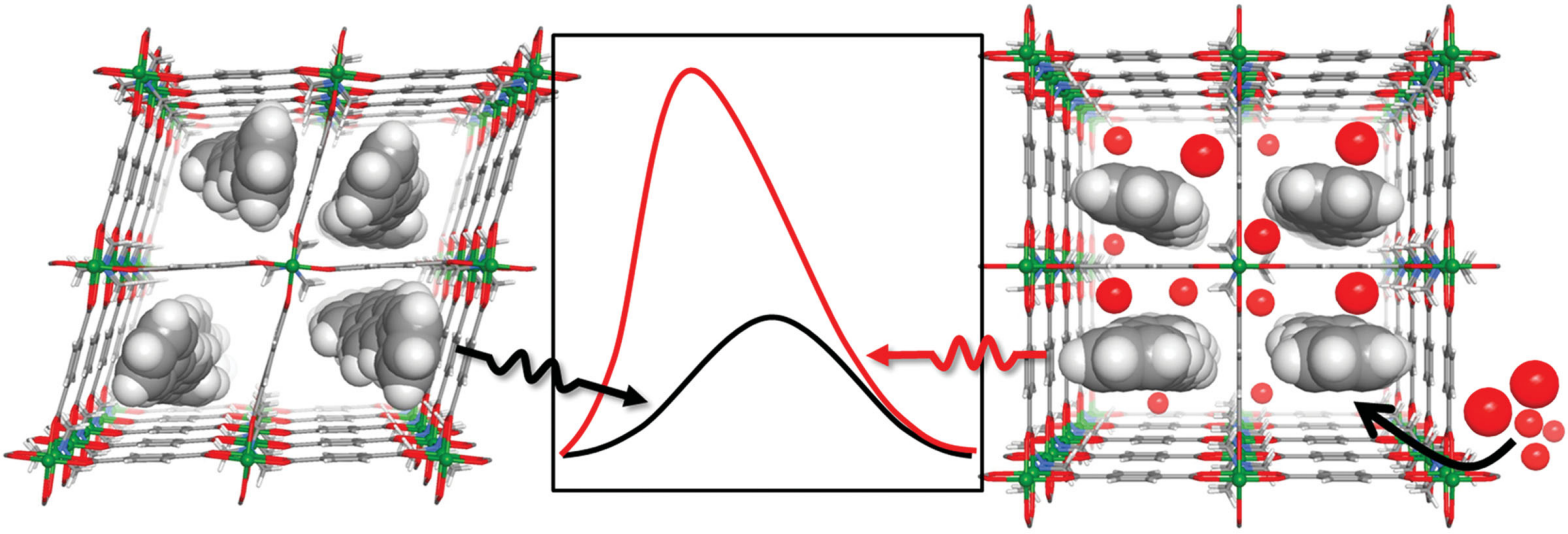
Structural transformation and luminescence change of the DSB molecules in DSB@[Zn_2_(bdc)_2_(dabco)] due to steric hindrance effect of the adsorbed gas molecules.

The structural transformation of a luminophore is not restricted in the host framework. Uemura and Kitagawa et al. reported an interesting strategy for luminescence detection of CO_2_ and C_2_H_2_ by utilizing the coupled structural transformations of a fluorescent guest and the host framework in [Zn_2_(bdc)_2_ (dabco)]·DSB (DSB@MOF, H_2_bdc = 1,4‐benzenediarboxylic acid, dabco = 1,4‐diazabicyclo‐[2.2.2]octane, DSB = distyrylbenzene) (**Figure** **2**).^82^ The host framework is a flexible jungle‐gym like structure, which possesses square 1D channels in its open form and squashed rhombic 1D channels in both the guest‐free and DSB‐included forms. DSB@MOF could selectively adsorb C_2_H_2_ and CO_2_ over other atmospheric gases, such as N_2_, O_2_, and Ar, inducing a host framework expanding accompanied by alteration of the DSB conformation. At 195 K, upon exposure to C_2_H_2_ above 7.0 kPa or CO_2_ above 30 kPa for a few minutes, the weak fluorescence of DSB@MOF (centered at about 485 nm) changed to a stronger blue fluorescence centered at about 425 nm, which can be ascribed to the conformational variations of DSB, leading to large changes in its fluorescence properties. The structural transformation of the MOF, although seemingly has no effect on the fluorescence change, should play an important role for selective adsorption of C_2_H_2_ over CO_2_, as well as stabilization of the specific conformations of the DSB molecules. Such fluorescence response is selective and reversible, which relies on a fluorescence change of organic molecules without any chemical interaction or energy transfer.

**Figure 3:**
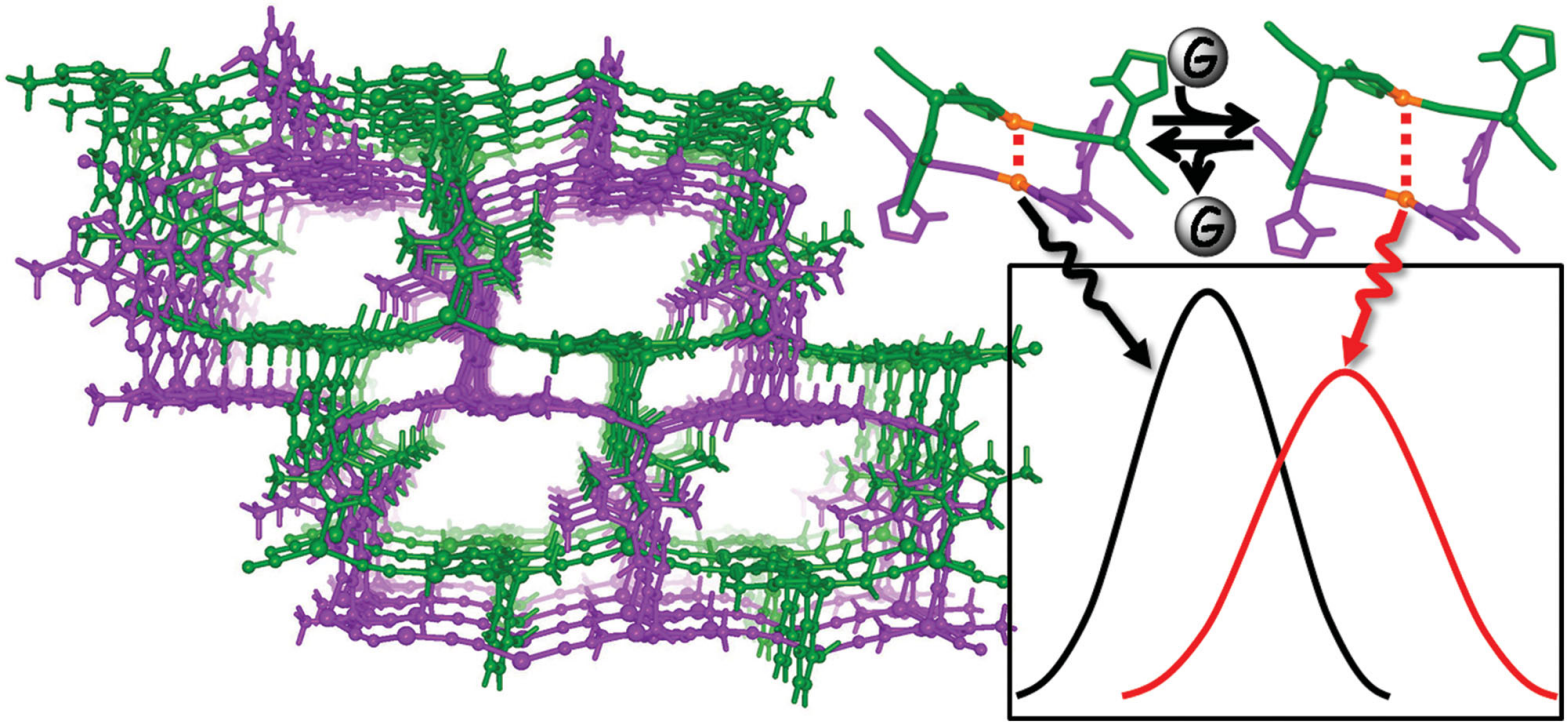
Structure of [(CuCN)_3_ (bdpzmpy)] and its guest‐dependent luminescence induced by change of interframework interactions.

More accurate structure‐property relationship can be obtained when single‐crystal structures of the guest‐included MOFs are available.^83^ Li et al. reported a luminescent MOF [(CuCN)_3_(H_2_ bdpzmpy)]·guest (bdpzmpy = 2,6‐bis((3,5‐di‐methyl‐1*H*‐pyrazol‐4‐yl)methyl)pyridine) showing reversible solvent‐responsive luminescence and real‐time response toward acetonitrile vapor (**Figure** **3**).^84^ This MOF is a two‐fold interpenetrated **srs** network consisting of 1D CuCN helical chains linked by bdpzmpy, retaining 1D open channels (ca. 5.5–7.8 Å). The as‐synthesized compound showed cyan luminescence centered at 490 nm, while its desolvated form exhibited blue luminescence centered at 450 nm. After immersion of the desolvated MOF in various organic solvents, the emission maximum showed 30–80 nm red‐shift. The guest‐dependent luminescence responses were attributed to different extents of metal‐to‐ligand or intra/interligand charge transfers as well as different Cu···Cu contacts induced by this flexible MOF, which were structurally confirmed by single‐crystal X‐ray diffraction study. Although desolvation and/or solvent‐exchange led to damage of single‐crystallinity, solvent‐inclusion single‐crystals can be obtained by direct syntheses from the corresponding solvents. For example, the intermolecular Cu···Cu separation changes 0.05 Å and the emission maximum changes 40 nm from the as‐synthesized to the acetonitrile‐saturated form. Upon exposing a thin film compressed from mixed powders of the desovlated MOF and KBr in acetonitrile vapor with continuously increasing concentration, the emission at 450 nm gradually decreased, and a new peak at around 530 nm appeared as a shoulder.

**Figure 4:**
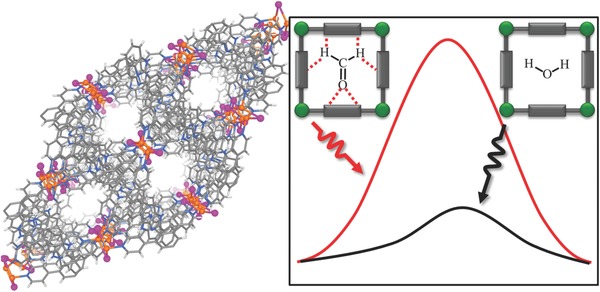
Structure of [Cu_2_(bimbpyb)_2_I_2_] and its luminescence enhancement upon replacing guest H_2_O with HCHO gas which rigidifies the host framework.

Some insignificant transformation of the host framework can also result in totally different sensing performance. Dong et al. reported a microporous [Cu_2_(bimbpyb)_2_I_2_]·4H_2_O (bimbpyb = 1‐benzimidazolyl‐3,5‐bis(4‐pyridyl)benzene) showing highly sensitive luminescence enhancement toward HCHO vapor, occurred in a single‐crystal‐to‐single‐crystal fashion (**Figure** **4**).^85^ Upon exposed [Cu_2_(bimbpyb)_2_I_2_]·4H_2_O to air containing trace HCHO, a luminescence enhancement with slightly blue‐shifted emission was observed, which was attributed to the structural rigidity enhancement imposed by the host‐guest interactions and the structural variations of the Cu_2_I_2_ core. The HCHO‐adsorbed crystal possesses a composition of [Cu_2_(bimbpyb)_2_I_2_]·2HCHO·H_2_O, with multiple hydrogen bonding between HCHO and the host framework, accounting for its high sensitivity. In contrast, much weaker interactions between H_2_O and the host framework were found in [Cu_2_(bimbpyb)_2_I_2_]·4H_2_O. Nevertheless, very small structural alterations of the host framework (<0.03 Å) were observed after the guest replacement. Therefore, the luminescence enhancement and blue shift was attributed to the strengthening of the host framework by HCHO.

**Figure 5:**
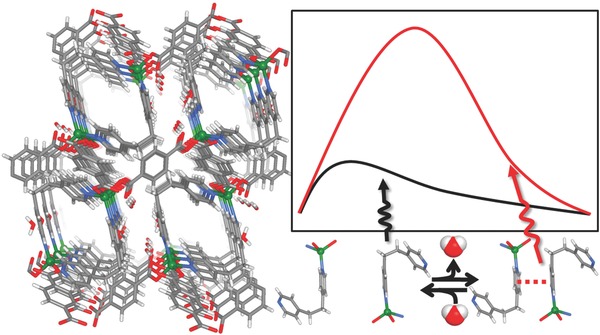
Structure of [Zn(dpe)(bdc)]·2H_2_O and its luminescence change upon adsorption and desorption of H_2_O vapor, which lead to weak and strong luminescence, because of long and short π–π interactions between the dpe ligands, respectively.

In addition, useful information of structural transformation can also be obtained by using powder X‐ray diffraction (PXRD), being favorable for understand the sensing behavior. Wang et al. reported a 3D MOF [Zn(dpe)(bdc)]·4H_2_O (dpe = 1,2‐bis(4‐pyridyl)ethane) which can show H_2_O‐dependent luminescence properties (**Figure** **5**).^86^ This MOF is 5‐fold interpenetrated diamondoid network filled with 2D water layer containing hydrogen‐bonded (H_2_O)_16_ rings. The as‐synthesized MOF showed a two‐step water removal with a reversible desolvated/resolvated process from 30 to 86 °C, giving a partially dehydrated phase [Zn(dpe)(bdc)]⋅2H_2_O, and an irreversible one from 195 to 250 °C. It showed reversible luminescent response to humidity upon cycling between room temperature (emission maxium at 470 nm) and 86 °C (emission maxium at 510 nm), giving a large red shift and significant intensity increase of the emission. The mechanism is considered to be shortening (0.03 Å) of the inter‐channel distance and enhancing the π–π stacking between two dpe moieties due to desorption of the intra‐channel water molecules, which was supported by crystal structure measurement from PXRD data of the partially dehydrated phase [Zn(dpe)(bdc)]·2H_2_O.

As shown by the above discussed examples, regardless of the host or guest luminescent centers, structural flexibility can usually result in significant luminescence changes. The luminescence emission wavelengths of flexible MOFs usually shift upon exposure to guest analytes (note that the emission intensities generally change simultaneously), because the guest‐induced structural transformations alter the separations and interactions between multiple luminophores.

## 3 Coordinative Guests

Many small molecules possessing O/N/S donor atoms can coordinate at the open metal sites (OMSs) on the pore surface of MOFs, which can facilitate non‐radiative relaxation of the excited state and lead to luminescence quench. The most representative examples are water induced luminescence quenching of lanthanide metal based MOFs,^87^ because their luminescence directly emit from the oxophilic lanthanide ions which have strong coordination ability for H_2_O,^88^, ^89^, ^90^ and the high‐energy O–H oscillators can effectively match electronic energy gaps of the lanthanide ions.^91^

**Figure 6:**
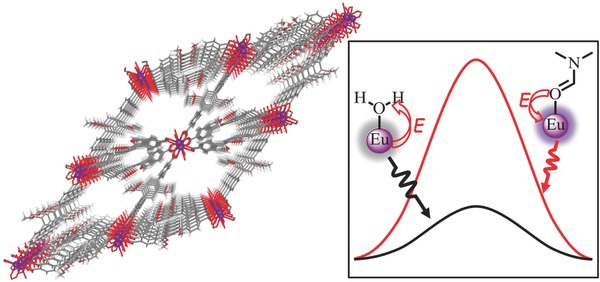
Structure of [Eu_2_(bmtpdc)_3_(H_2_O)_4_] and illustration of its luminescence enhancement mechanism, in which DMF vapor replace the coordinated H_2_O ligand that strongly quenches the Eu(III) luminescence.

The luminescence quenching effect of water coordination can be utilized for detection of other coordinative guests, in which the coordinated water molecules are replaced by other guest with weaker quenching effect, leading to a luminescence enhancement. For example, Song et al. reported a lanthanide MOF [Eu_2_(bmtpdc)_3_(H_2_O)_4_]·3DMF (H_2_bmtpdc = 2′,5′‐bis(methoxymethyl)‐[1,1′:4′,1′′‐terphenyl]‐4,4′′‐dicarboxylic acid, DMF =*N*,*N*‐dimethylformamide) exhibiting selectively “turn‐on” luminescence response for DMF vapor (**Figure** **6**).^92^ This MOF is a 3D framework structure consisting of 1D Eu_2_(RCOO)_6_(H_2_O)_4_ infinite rods and dicarboxylate pillars, with 3D channel occupied by the DMF guest molecules. The characteristic Eu(III) luminescence of the as‐synthesized MOF reduces ca. 32% after the guest DMF molecules were replaced by water upon solvent‐exchanged treatment. When the H_2_O‐exchanged MOF was exposed in saturated vapors of various organic solvents, a more than 8‐fold enhancement of luminescence was observed for DMF, while most of the other solvents gave only around 1‐fold enhancement. To obtain reliable luminescence intensities, the sample holder was quickly transferred between the vapor container and spectrometer, and each measurement was repeated for many times. Noteworthily, PXRD patterns of the as‐synthesized sample and its H_2_O‐exchanged state are very similar, indicating little framework distortion. However, exposure of the H_2_O‐exchanged sample to DMF vapor resulted in significant change of PXRD pattern or obvious framework distortion, indicating that DMF substitutes coordinated water and leads to the turn‐on response. The strong turn‐on response to DMF was further attributed to the more efficient ligand to metal charge transfer (LMCT) process, caused by DMF–ligand interactions that presumably shift the excited state energy level of the ligand.

**Figure 7:**
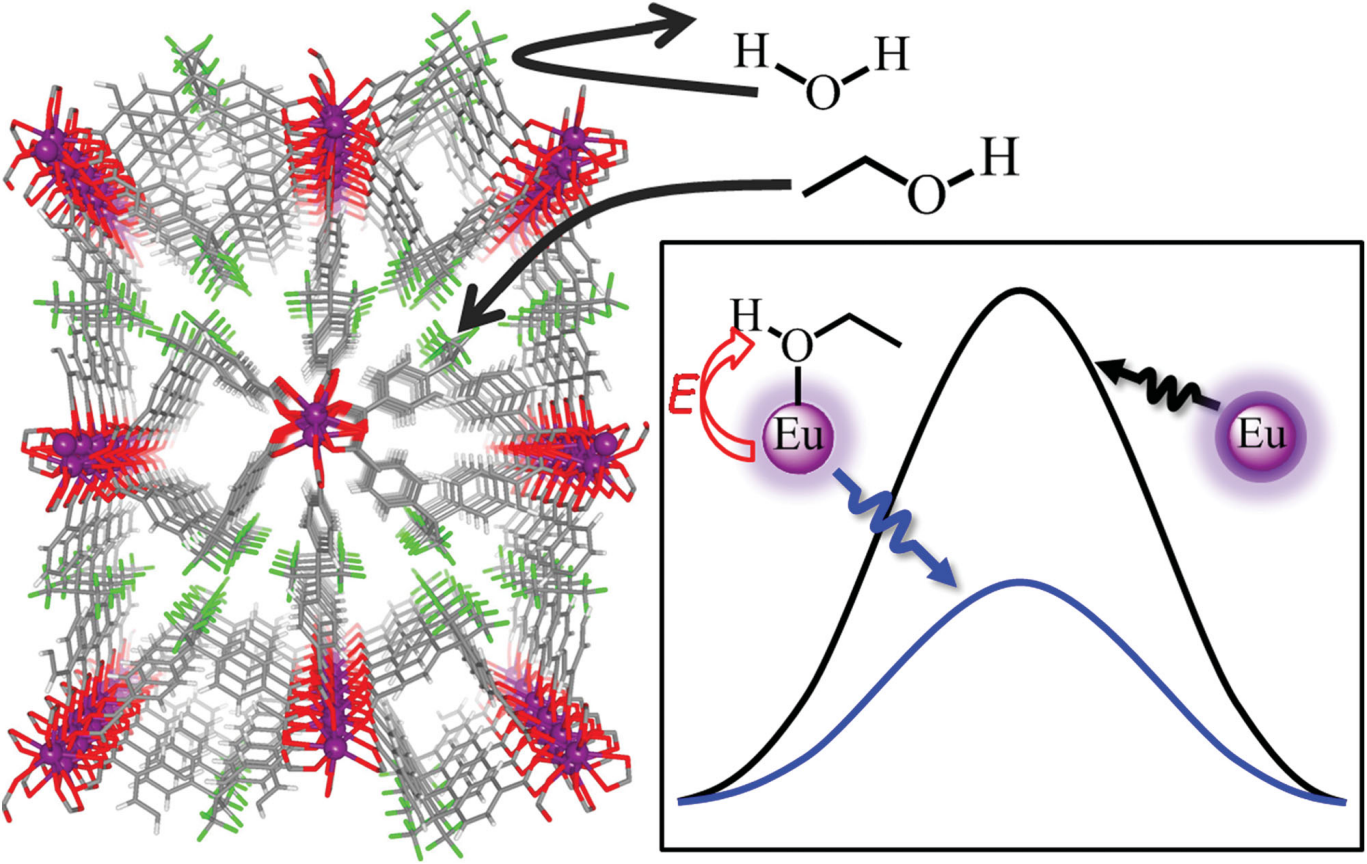
Structure of [Eu_2_(hfipbb)_3_] and illustration of its ethanol vapor selective luminescence quenching mechanism over water vapor.

Compared with other molecules, the small H_2_O molecules have stronger coordination ability and quenching effect for lanthanide complexes. However, with a suitable pore structure, a lanthanide MOF can achieve selective luminescence sensing of other molecules over H_2_O. For example, Corma et al. reported a hydrophobic MOF [Eu_2_(hfipbb)_3_]·*x*DMF (ITQMOF‐1‐Eu, H_2_hfipbb = 4,4′‐(hexafluoroisopropylidene)bis(benzoic acid)) (**Figure** **7**).^93^ The structure of this compound was not directly determined because the crystals exhibit serious twinning. Instead, another related structure possessing the same chemi­cal composition was determined by single‐crystal X‐ray diffraction as infinite zigzag rod‐shape Eu_2_(RCOO)_6_ chains linking by hfipbb^2−^. Nevertheless, the N_2_ isotherm at 77 K showed that the pore volume of guest‐free ITQMOF‐1‐Eu is 0.14 cm^3^ g^−1^, and the Brunauer–Emmett–Teller (BET) surface area is 207 m^2^ g^−1^. Under alternating streams of ethanol‐saturated and ethanol‐free air, the characteristic emission of ITQMOF‐1‐Eu showed a reversible and rapid luminescence quenching toward ethanol. The sensing mechanism was rationalized by considering that ethanol may coordinate to the Eu(III) ions, through coupling with the vibrational states of the O–H oscillators. More interestingly, the luminescence of this material was not quenched by water, so that it can efficiently sense ethanol in the presence of water, which was ascribed to the hydrophobic ligand hfipbb^2–^ and pore surface.

Other coordinative gas can have stronger quenching effect compared with water vapor.^94^ For example, Humphrey et al. reported that [Tb(tctpo)(H_2_O)]·2DMF·H_2_O (PCM‐15, H_3_tctpo = tris(*p*‐carboxyl)triphenylphosphine oxide) can be used to quantitatively detect trace amounts of NH_3_.^87^ To obtain reliable luminescence intensity changes, a custom cell was designed to be directly interchangeable between the gas adsorption analyser and the spectrophotometer, which allowed each sample of PCM‐15 to be activated under vacuum, directly exposed to adsorbates in situ, and studied spectrophotometrically over many cycles without physical manipulation or exposure to air, and each measurement was repeated three or more times using freshly prepared samples to provide error ranges. Fifteen gases (1 atm) and vapors (saturated) were used to study their abilities to quench the Tb(III) luminescence of the fully desolvated MOF, among which NH_3_ was found to show the highest quenching efficiency of 60%, while H_2_O showed only 44% quenching. The quenching mechanism was attributed to the coordination of NH_3_ on the Tb(III) open metal sites. Compared to O–H oscillators in H_2_O, the increased number of N–H oscillators per NH_3_ molecule bound to the Tb(III) ions accounted for higher quenching efficiency.

**Figure 8:**
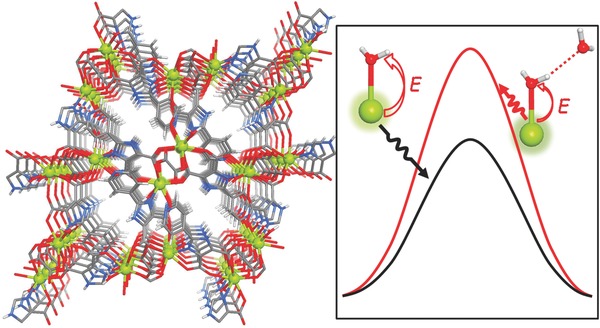
Structure of [Ln(pyimdc)(ox)_0.5_(H_2_O)] and illustration of its luminescence enhancement mechanism toward H_2_O vapor, via hydrogen bonding interaction with the H_2_O ligand, which reduces the frequency of the O–H vibration of the H_2_O ligand.

In some cases, as an analyte, water vapor may enhance rather than quench the luminescence intensity of lanthanide MOFs, because it reduces the quenching effect of a water ligand. For example, Dong et al. reported two lanthanide‐based MOFs [Ln(pyimdc)(ox)_0.5_(H_2_O)]·2H_2_O (Ln(III) = Eu(III), Tb(III), H_2_pyimdc = pyridyl‐4,5‐imidazole dicarboxylic acid, H_2_ox = oxalic acid) with turn‐on luminescence responses toward humidity (**Figure** **8**).^95^ These MOFs contain ellipse‐like channels with the dimensions of 8.9 × 7.5 Å^2^, being filled by (H_2_O)_4_ clusters that interacting with the coordinated H_2_O on Ln(III). Interestingly, when the MOFs were heated at 200 °C for 2 hours, only two guest water molecules were removed, while the coordinated water molecule was retained. After that, the characteristic luminescence of these MOFs showed a decrease in luminescence emission intensity compared with their as‐synthesized state. Therefore, these partially dehydrated MOFs can be used to sense water vapor with a turn‐on mechanism, in which hydrogen bonding interactions between the O–H moieties of the coordinated H_2_O molecule and the electron lone pair of the analyte H_2_O to reduce the O–H vibration frequency and quenching effect of the H_2_O ligand.

**Figure 9:**
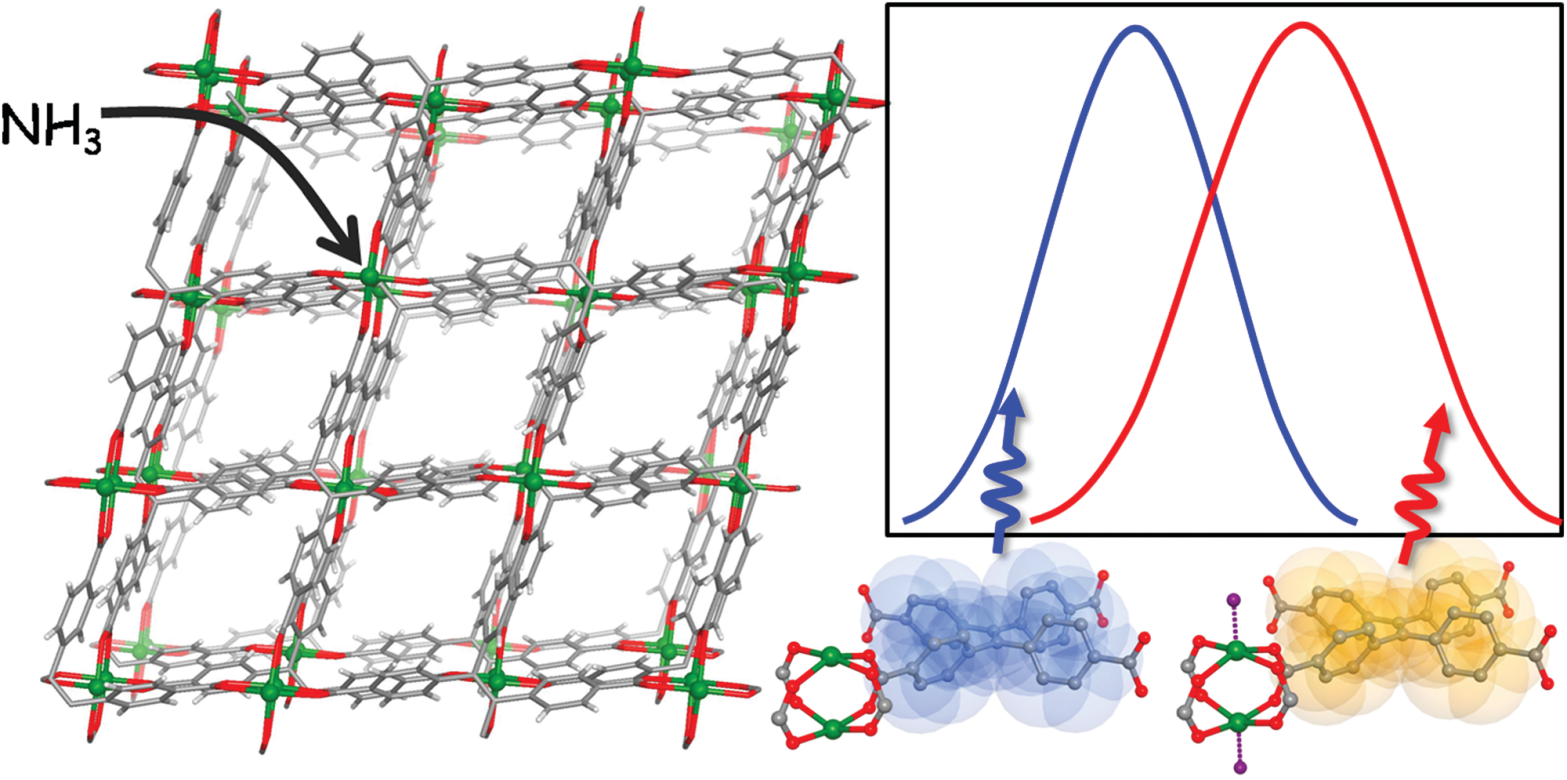
Structure of [Zn_2_(tcpe)] and illustration of its luminescence bathochromic effect via coordination with NH_3_ gas.

The gas coordination effect can not only quench the luminescence of the lanthanide metal ions but also perturb the excited state energy of the organic fluorophore.^96^, ^97^ For example, Dincaˇ et al. reported two MOFs [Zn_2_(tcpe)(H_2_O)_2_ ]·4DEF and [Mg(H_2_dhbdc)(DMF)_2_] (H_4_tcpe = tetrakis(4‐carboxyphenyl)ethylene, DEF = *N*,*N*‐diethylformamide, H_4_dhbdc = 2,5‐dihydroxybenzene‐1,4‐dicarboxylic acid) showing shifts of emission wavelengths upon interaction with ammonia at 100 °C (**Figure** **9**).^96^ [Zn_2_(tcpe)] shows staggered 2D layers constructed from paddle‐wheel shaped Zn_2_(RCOO)_4_ clusters linked by tcpe^4−^ ligands, and is permanently porous with BET surface area of 317 m^2^ g^−1^. [Mg(H_2_dhbdc)] is constructed from 1D Mg_2_(RCOO)_4_ chain linking by H_2_dhbdc^2−^ linkers in an alternative manner, showing 1D channel about 5.3 × 5.3 Å^2^. Both fluorescent MOFs maintain their luminescence above 100 °C. Exposure of activated [Zn_2_(tcpe)] to triethylamine, ethylenediamine, DEF, and water vapors, as well as ammonia gas at room temperature shifted its emission maximum by 0−23 nm. Interestingly, only ammonia exposure (1% ammonia in nitrogen) is able to shift the emission maximum at 100 °C. Computational simulation revealed that such temperature‐dependent luminescence response may be attributed to the stronger binding of NH_3_ to the OMSs. While the NH_3_ sensing of [Zn_2_(tcpe)] is irreversible, [Mg(H_2_dhbdc)] showed a reversible but smaller fluorescence response to ammonia at 100 °C, which could be attributed to the weaker coordination ability of Mg(II) toward NH_3_.

The coordination effects of guests can be rationally used to modify the luminescence property and guest‐responsive property of MOF crystals. For example, Balkus et al. fabricated an orientated thin film (ca. 3 μm thick) of a fluorescent MOF [Zn_2_(bpdc)_2_ (bpee)]·2DMF (LMOF‐111, H_2_bpdc = 4,4′‐biphenyldicarboxylic acid, bpee = 1,2‐(bipyridyl)ethene)^98^ on a glass substrate, and then immersed it in a DMF solution of AgNO_3_ to obtained a Ag(I) functionalized LMOF‐111 thin film.^99^ LMOF‐111 is a 3D coordination network consisting of undulating Zn_2_(bpdc)_2_ layers and bpee pillars, as well as roughly rectangular 1D channels with pore‐size distribution around 7.5 Å.^100^ AgNO_3_@LMOF‐111 showed weaker fluorescence than the pristine material LMOF‐111, which was attributed to the coordination of Ag(I) with the C=C bond of the bpee linker. Upon exposure to propylene, the emission intensity of AgNO_3_@LMOF‐111 was enhanced by ca. 43%, while a fluorescence quenching of 12% was observed when exposed to propane gas. Such a fluorescence enhancement was attributed to the weakening of the Ag(I)‐bpee coordination after the formation of additional coordination bonds between the alkene analyte and Ag(I). The sensing response to propylene was reversible after heating at 60 °C under vacuum followed by nitrogen purging.

MOFs containing OMSs can bind strongly with coordinative guests, being favorable for improving the sensitivity but disadvantageous for improving the selectivity (difficult to distinguish different coordinated guests). In this context, water vapor which commonly exists in air should be the main concern because of its small size and strong coordination ability with OMSs. Fortunately, as shown by the above discussed examples, coordinative guests can change the luminescence properties of MOFs by many ways, and the water effect could be avoided or even utilized by rational consideration of the pore structures and luminescence mechanisms of MOFs.

## 4 Nitro‐Containing and Aromatic Compounds

**Figure 10:**
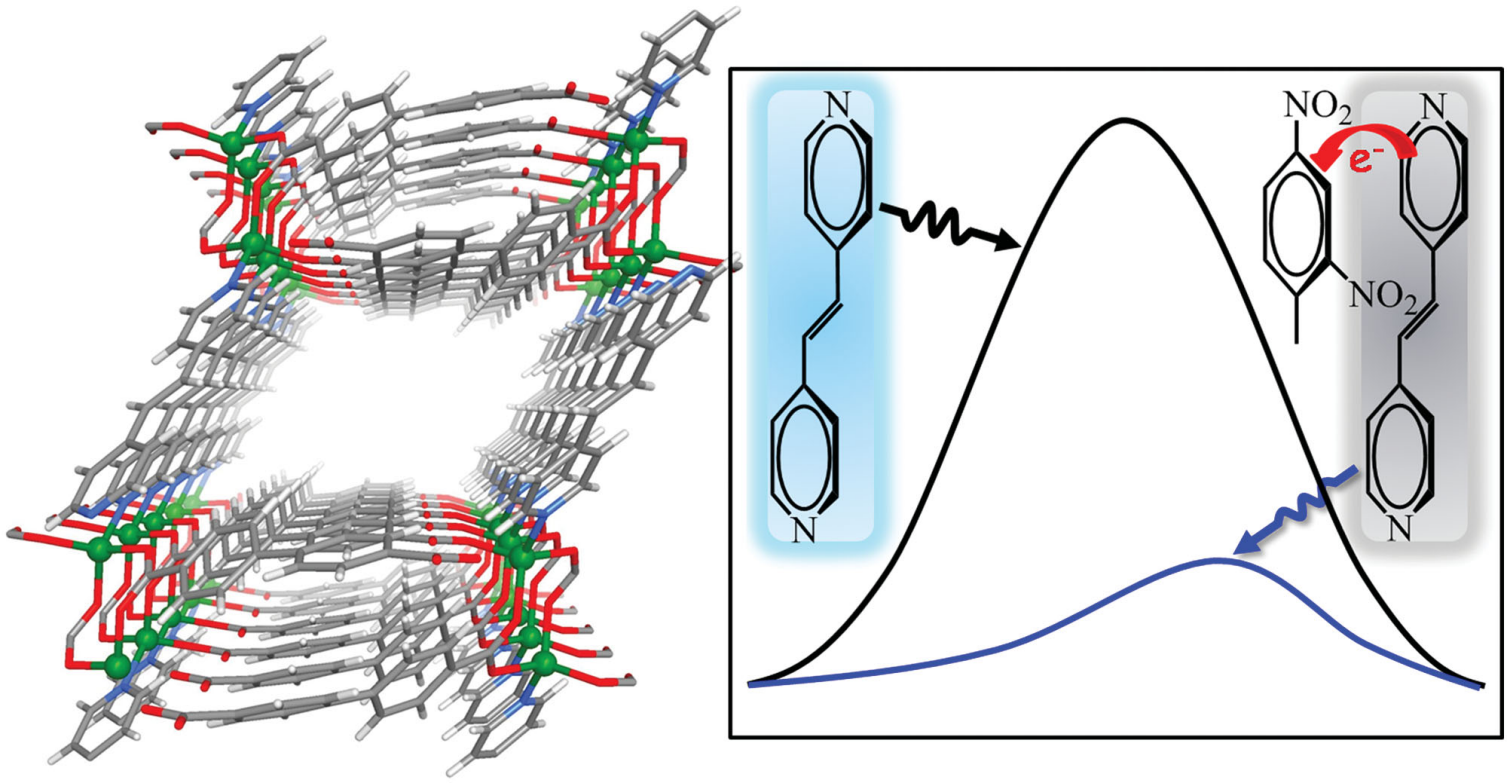
Structure of [Zn_2_(bpdc)_2_(bpee)] and its luminescence quenching due to electron transfer from the organic fluorophore to DNT.

Organic molecules containing nitro groups are well known for their electron‐deficient nature and strong luminescence quenching effect, which can be beneficial for detection of many nitro‐containing explosives.^101^, ^102^, ^103^, ^104^, ^105^, ^106^, ^107^, ^108^, ^109^ Li et al. demonstrated the first application of luminescent MOFs in detection of nitro explosives.^100^ The guest‐free LMOF‐111 (**Figure** **10**) exhibited fluorescence emission centered at 420 nm upon excitation at 320 nm, which was assigned to the organic linkers. To study the luminescence sensing behavior of LMOF‐111 toward nitro explosive 2,4‐dinitrotoluene (DNT) and the plastics explosive taggant 2,3‐dimethyl‐2,3‐dinitrobutane (DMNB), ground powders of LMOF‐111 were glued onto glass slides. The fluorescence emission was quenched more than 80% within 10 seconds, accompanying with 42 nm red‐shift of the emission maximum when the MOF was exposed in saturated vapors of DNT (0.019 Pa) or DMNB (0.29 Pa) in air, which shows a higher sensitivity and faster response than conventional luminescence sensing materials such as conjugated polymers (20–40% quench after 10–20 seconds exposure). In addition, the original fluorescence of the MOF can be recovered by heating at 150 °C. Similar with other fluorescence probes, the fluorescence responses of LMOF‐111 were attributed to electron transfer from the excited host framework to the electron deficient DNT and DMNB molecules. Because DMNB is generally difficult to detect due to the lack of π–π interactions with the host framework, the remarkable fluorescence sensitivity of LMOF‐111 was further attributed to the pore confinement effect of the molecular‐sized channel which facilitates stronger interactions between the explosives and the host framework.

**Figure 11:**
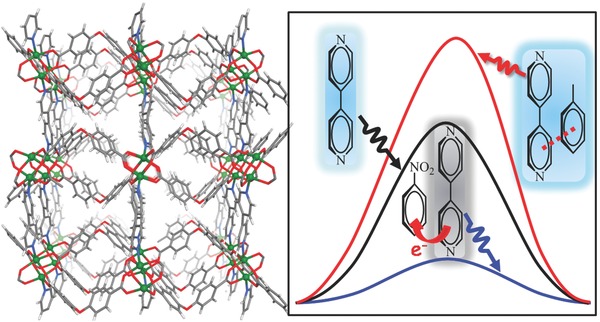
Structure of [Zn_2_(oba)_2_(44bpy)] and its luminescence quenching and enhancement mechanisms toward electron deficient and electron rich molecules exemplified by nitrobenzene and toluene, due to electron transfer and exciplex formation, respectively.

The fluorescence quenching ability of a guest may depend not only on its electron withdrawing ability but also other factors. For example, Li et al. reported a MOF [Zn_2_(oba)_2_(44bpy)]·*x*DMA (LMOF‐121, H_2_oba = 4,4′‐oxybis(benzoic acid), 44bpy = 4,4′‐bipyridine, DMA =*N*,*N*‐dimethylacetamide) exhibiting different fluorescence quenching and enhancement efficiencies toward a series of nitro‐containing and aromatic volatile organic compounds (**Figure** **11**).^110^ LMOF‐121 is a 2‐fold interpenetrated 3D network containing 1D channels with size ca. 5.8 × 8.3 Å^2^. Upon excitation at 280 nm, the guest‐free compound emits ligand centered fluorescence with maximum at 420 nm. Ground powders of LMOF‐121 were glued onto glass slides for luminescence sensing. When exposed to saturated vapors of nitro‐containing aromatic analytes, the fluorescence was quenched to varying degrees following an order of nitrobenzene >*m*‐dinitrobenzene > nitrotoluene ≈ *p*‐dinitrobenzene > DNT, and can be fully recovered by heating at 150 °C for a few minutes. Besides the different electron‐withdrawing abilities of the analytes, other factors must be involved for the observed trend. For example, the strongest and relatively poor quenching effects of nitrobenzene (32 Pa) and *m*‐/*p*‐dinitrobenzene (0.12/0.0032 Pa), respectively, which violated their electron withdrawing abilities, was attributed to their significantly different saturation vapor pressures. Notably, LMOF‐121 can selectively detect aromatic DNT over aliphatic DMNB (quenching efficiency < 1%). Besides the weak ability for electron acceptance, DMNB (7.1 × 7.3 × 7.7 Å^3^) is also too large to enter the smaller pore of the MOF.

As an opposite effect, electron‐rich aromatics enhanced the luminescence with an order of toluene > benzene > chlorobenzene. The fluorescence quenching/enhance mechanism was explained by the excited state electron transfer process, according to the reduction potential measurements and computational studies. For an electron deficient analyte, electrons transferred from the conduction band of LMOF‐121 to the lowest unoccupied molecular orbital (LUMO) of the analyte upon excitation, followed by a non‐radiative relaxation.^110^ For an electron‐rich analyte, an opposite electron transfer process was proposed. However, a complete electron transfer is not likely, because this behavior would instead cause non‐radiative relaxation of the excited state. Therefore, a more plausible explanation is that the analyte inhibits linker motions (at the excited state),^2^ or formation of linker‐analyte exciplex.

The direct detection of some nitro explosives can become enormously difficult due to their extremely low vapor pressures. The fluorescent MOF [Zn_2_(hfdc)_2_(44bpy)]·*x*DMA (LMOF‐202, H_2_hfdc = 9*H*‐fluorene‐2,7‐dicarboxylic acid) is a 2‐fold interpenetrated **pcu** network containing 3D intersecting channels.^111^ Desolvation of LMOF‐202 leads to a structural change as indicated by PXRD, and the guest‐free MOF [Zn_2_(hfdc)_2_(44bpy)] is porous with BET surface area of 136 m^2^ g^−1^ and displays significantly red‐shifted (60 nm) fluorescence centered at around 490 nm. Exposure to the saturated vapors of various ketones, the emission intensity of guest‐free [Zn_2_(hfdc)_2_(44bpy)] obviously enhanced, accompanying with small blue‐shifts in emission wavelength. These fluorescence responses were ascribed to exciplex formation between ketones and MOF. However, considering that the π‐conjugation systems of ketones are too small, and the guest‐modulated fluorescence occurs at shorter wavelengths, a steric hindrance effect, i.e., the guest restricts the flexibility/motion of the host framework which reduces non‐radiative relaxation, might be more plausible.^2^, ^112^ Importantly, the fluorescence sensing of cyclohexanone can be used to indirectly detect 1,3,5‐trinitroperhydro‐1,3‐5‐triazine (RDX), because the vapor pressure of RDX (6 × 10^−7^ Pa) is too low to detect and cyclohexanone coexists in RDX as recrystallization solvent.^111^ Further exposure to a sample of RDX recrystallized in cyclohexanone resulted in a >12% enhancement of emission intensity within 15 minutes, demonstrating the feasibility of such indirect detecting of RDX.

**Figure 12:**
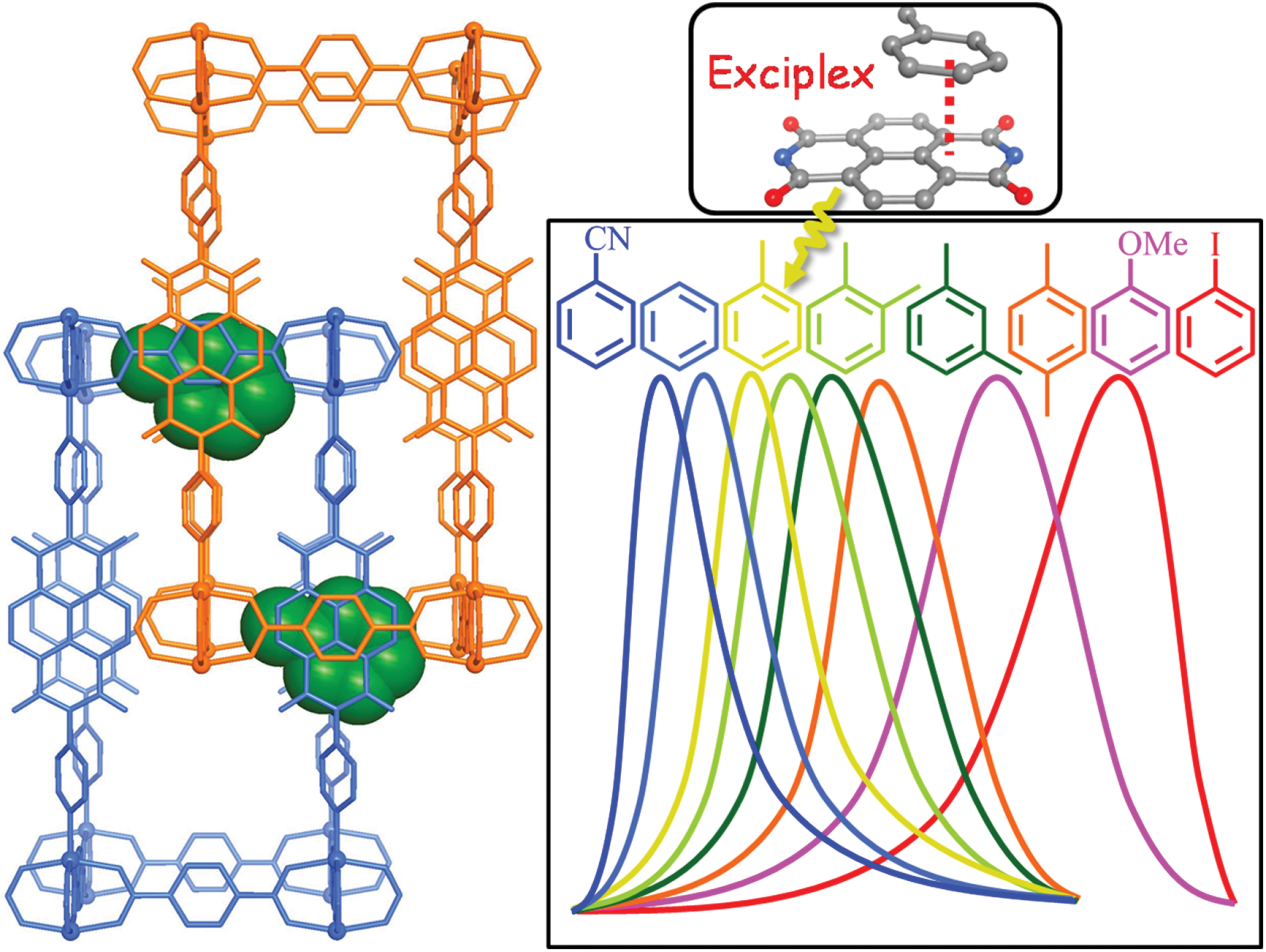
Structure of [Zn_2_(bdc)_2_(dpndi)]·2.5C_7_H_8_ (toluene molecules in the framework are highlighted in green with the space‐filling mode) and its guest‐dependent luminescence color via formation of exciplex between the ndi core and the aromatic guest with different charge‐transfer degrees, controlled by the ionization potentials of the guests.

Electron‐rich aromatic compounds can usually form exciplex with the fluorophores in MOFs to enhance fluorescence intensity.^107^ For example, 1,4,5,8‐naphthalenediimide (ndi) is known to generate exciplex emission when interacting with aromatic molecules. Kitagawa et al. reported an ndi‐based luminescent MOF [Zn_2_(bdc)_2_ (dpndi)]·4DMF (dpndi = N,N′‐di(4‐pyridyl)‐1,4,5,8‐naphthalenediimide),^113^ which is a flexible 2‐fold interpenetrated **pcu** network with changeable pore sizes. This MOF showed different emission wavelengths after the accommodation of a range of benzene derivatives with different ionization potentials, which was attributed to the formation of exciplex between the ndi core and the aromatic guest with different charge‐transfer degrees. However, it is worth noting that after a complete charge transfer, such as using *N*,*N*‐dimethylaniline as a guest, the pale‐yellow MOF crystal turned deep purple with completely quenched fluorescence due to the formation of a radical ion pair state between *N*,*N*‐dimethylaniline and ndi. While the as‐synthesized and guest‐free forms of the MOF both showed very weak fluorescence (quantum yield < 0.01, lifetime: 150 ps), the toluene‐saturated sample showed very high fluorescent intensity centered at 476 nm (quantum yield = 0.22, lifetime: 14.8 ns). Single‐crystal X‐ray structure of the toluene‐saturated phase [Zn_2_(bdc)_2_(dpndi)]·2.5C_7_H_8_ confirmed strong face‐to‐face π–π stacking interaction (3.6 Å) between the guest and the ndi core (**Figure** **12**). The luminescence response of this MOF was further tested in different toluene vapor pressures (0–90% of the saturation vapor pressure) by using a fluorescent microscope combining with a vapor‐control system (by mixing helium flow saturated with toluene and pure helium flow in different ratios), for which the powdered sample was packed in a gas‐flowing glass capillary. A nonlinear fluorescence enhancement effect against toluene vapor pressure was observed, which was attributed to the nonlinear adsorption amount against pressure and cooperative structural transition of the host.

The strong abilities of nitro‐containing and aromatic compounds for involving in electron or energy transfer processes at the excited states are not only beneficial for effective detection of these molecules, but also challenges for identification of a specific analyte from a mixture of similar compounds. Thus, for highly selective and specific explosive detection, MOFs with more accurate molecular recognition are required.

## 5 Radical Gases (O_2_)

Besides electron deficient molecules, unpaired electrons also have strong luminescence quenching effect by accepting energy from the excited‐state luminophores.^114^, ^115^, ^116^ For gases, NO and O_2_ are typical radical/paramagnetic molecules with a triplet ground state. These light gases, even when interact weakly with other substances in the structural point of view, can be efficiently detected by luminescence quenching.

For luminescence O_2_ sensing, the sensitivity is mainly determined by the O_2_ permeability, the original luminescence lifetime, and the collision radius of the luminescent dye.^117^, ^118^, ^119^, ^120^ Precious metal (Pt, Ru, Au, Ir, Re, etc.) complexes are widely used for O_2_ sensing due to their long phosphorescence lifetimes, being suitable for detecting O_2_ with extremely low concentrations.^121^ On the other hand, other luminescent probes with low sensitivities are useful for measuring high concentrations of oxygen. Because air pressure and wind speed change the partial pressure of O_2_, real‐time two‐dimensional image of air‐pressure or wind‐speed distribution can be obtained based on luminescence quenching of low‐sensitivity probes such as pyrene. However, these materials must be dispersed in gas permeable porous matrixes, usually organic polymers, to allow effective interaction with oxygen molecules.^120^ Because MOFs can have large porosity and various luminescence properties, they are very attractive for O_2_ sensing, and a number of advances have been reported in the past few years (**Table** **1**). It is worth noting that it is relatively easy to control the oxygen pressure, thus it is possible to measure the fluorescence intensity in situ at a range of oxygen pressures, giving a lot of useful information.

**Table 1:**
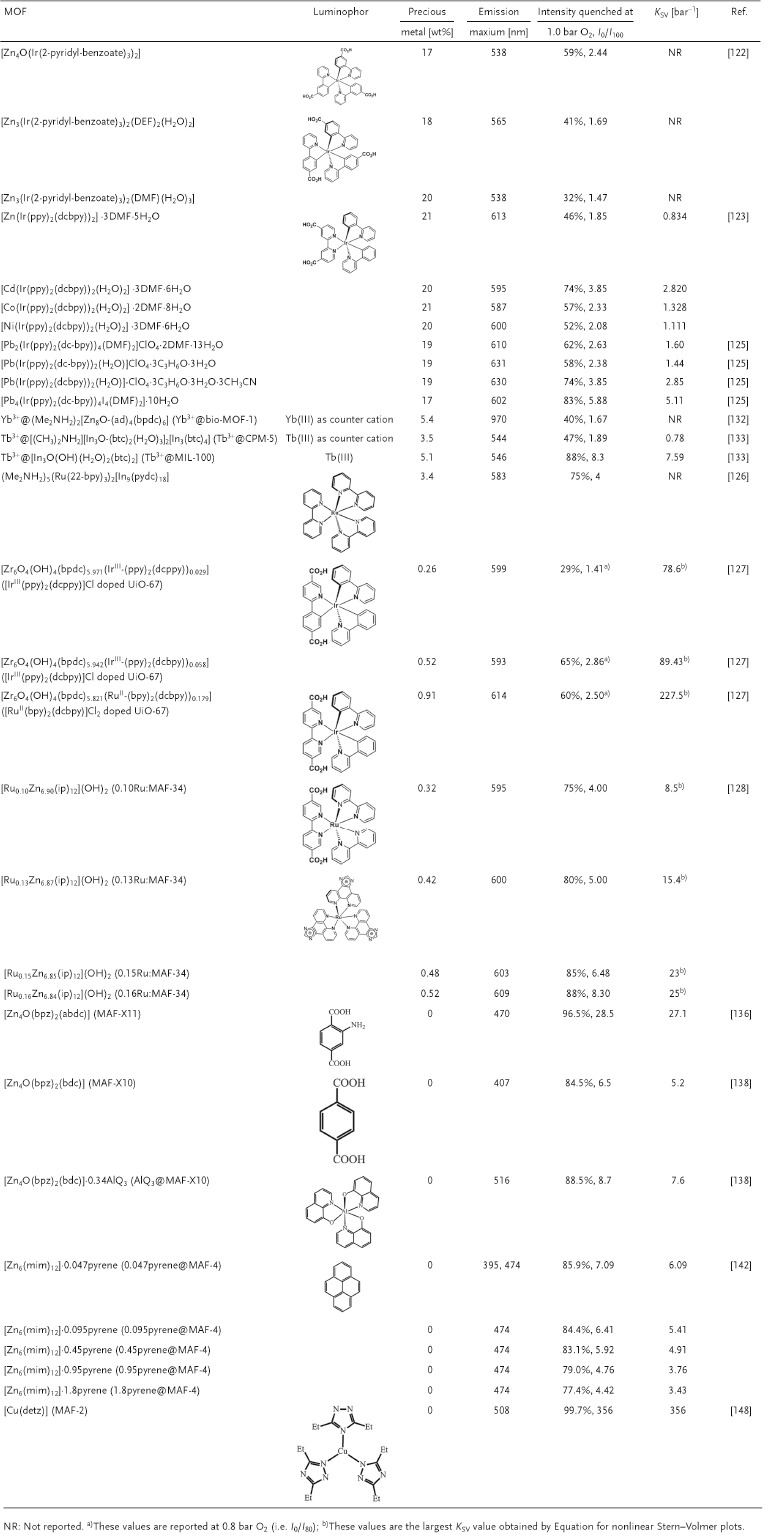
Comparison of the key parameters of known oxygen‐sensing luminescent MOFs

**Figure 13:**
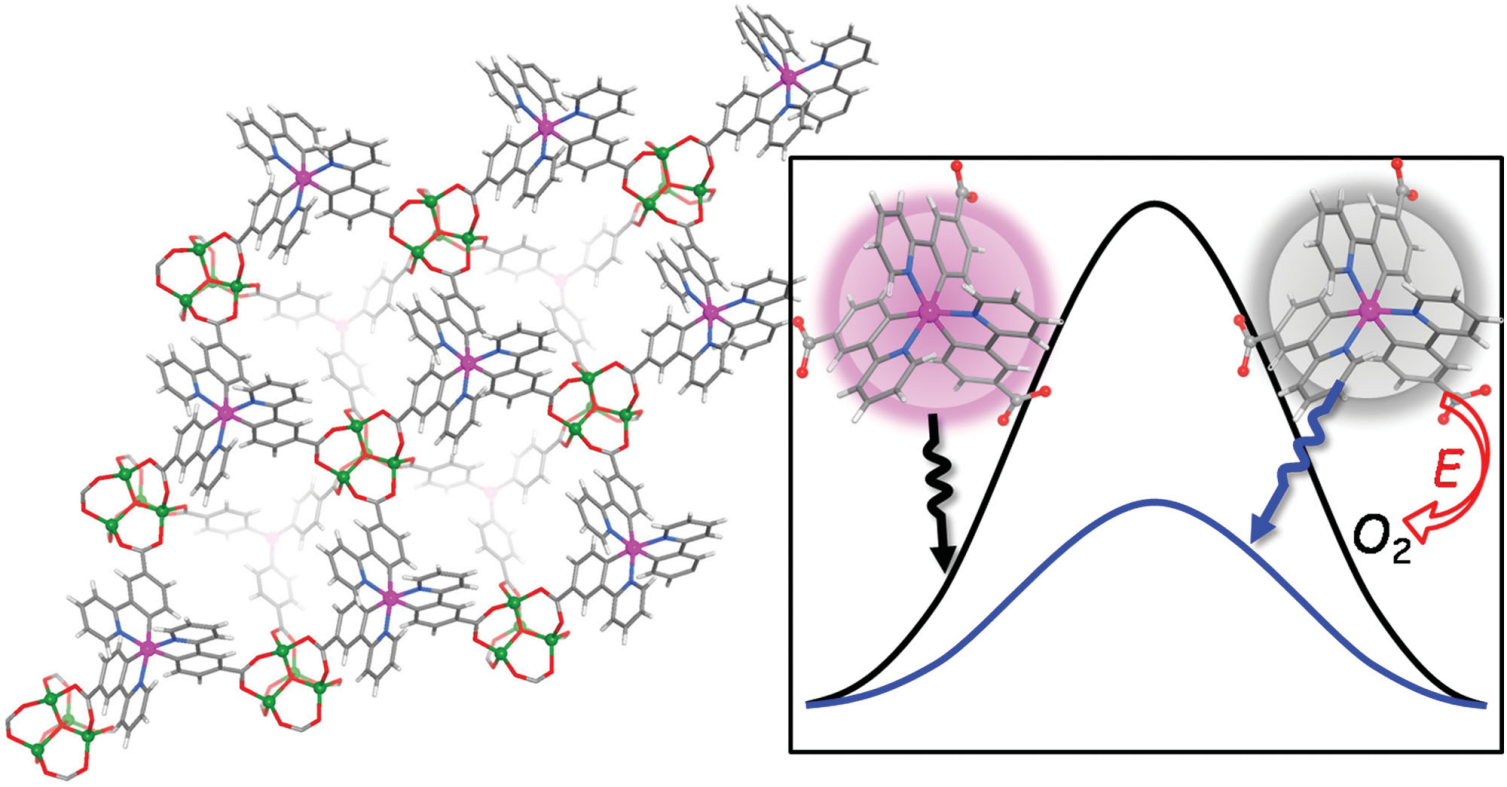
Structure of a bilayer MOF consisting of Zn_4_(μ_4_‐O)(RCOO)_6_ clusters and precious metalloligands derived from Ir(ppy)_3_ (Zn and Ir atoms are shown in green and pink, respectively), and luminescence quenching by O_2_ via energy transfer.

Lin et al. reported the first luminescent MOFs for O_2_ ‐sensing, by using bridging ligands derived from classic phosphorescent precious metal complexes. By connecting Zn(II) with two metalloligands derived from a common O_2_ sensitive complex Ir(ppy)_3_ (ppy = 2‐phenylpyridine), three phosphorescent MOFs were synthesized.^122^ Among the three MOFs, a 2D bilayer structure containing the classic Zn_4_O(RCOO)_6_ clusters and open channels of 7.9 × 4.3 Å^2^ is relatively robust with a large BET surface area of 958 m^2^ g^−1^ after desolvation (**Figure** **13**), while the other two containing both mononuclear and dinuclear Zn connecting nodes are nonporous toward N_2_ adsorption after activation though they both contain guest mole­cules at the as‐synthesized states. At 1 bar O_2_, the luminescence quenching efficiencies for the three MOFs and the two ligands are in the range of 32–59% and 8–16%, respectively, in which the tetranuclear‐based structure showing the highest efficiency of 59%. Moreover, only the tetranuclear‐based bilayer structure showed some cycling stability of luminescence upon repeated O_2_ dosing and removal. The distinct performance of this structure was ascribed to the permanent porosity which allows the collision of framework and O_2_ molecule.

Later, several similar precious metalloligands were also used to connect d^10^ transition metal ions or main group metal ions to form many other low‐dimensional coordination polymers containing solvent accessible voids for O_2_ sensing, in which their phosphorescence can be quenched by 46–83% at 1 bar O_2_ or *I*_0_/*I*_100_ = 1.85–5.88.^123^, ^124^, ^125^ Sun et al. also showed that [Ru(22bpy)_3_]^2+^ can be encapsulated as counter cation in an anionic coordination framework to give an O_2_‐sensitive (ca. 75% quenching at 1 bar O_2_) phosphorescent MOF (Me_2_NH_2_)_5_(Ru­(22bpy)_3_)_2_[In_9_(pydc)_18_]·4DMF·18H_2_O (H_2_pydc = pyridine‐2,5‐dicarboxylic acid).^126^ Interestingly, they can precisely control the hydrolysis of DMF by reaction time to obtain an isostructural non‐luminescent MOF with only Me_2_NH_2_^+^ counter cation, and even obtain a core‐shell structure with the phosphorescent one covered by the non‐luminescent analog. The core‐shell structure was insensitive to O_2_ because the outer shell prevents the core crystal to directly contact with O_2_.

**Figure 14:**
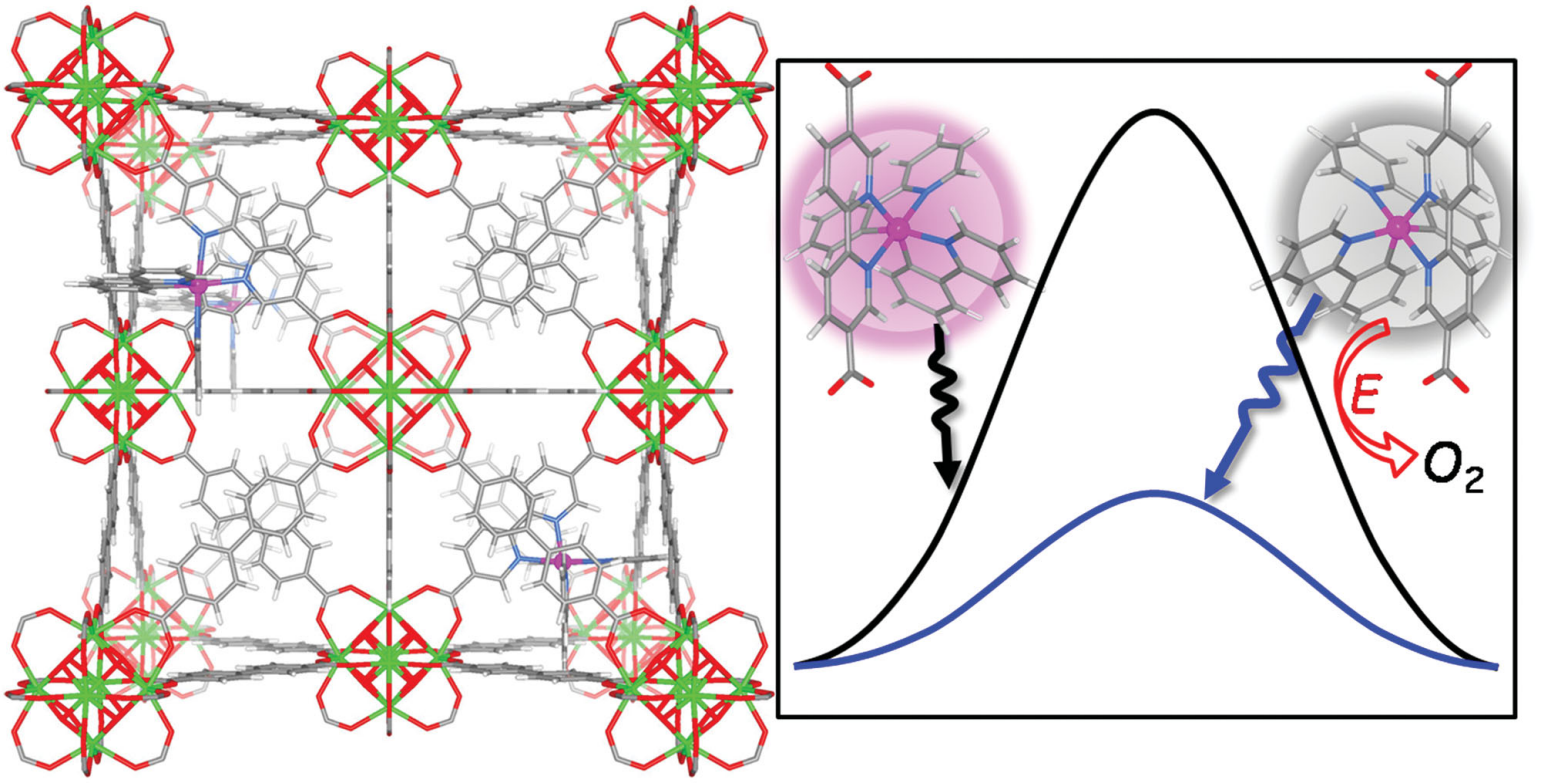
Structure of [Zr_6_O_4_(OH)_4_(bpdc)_6_] doped with Ir(ppy)_2_(22bpy) derived bridging ligands (Zr and Ir atoms are shown in bright green and pink, respectively) and its luminescence quenching by O_2_ via energy transfer.

Although MOFs constructing by precious metalloligands can be used directly for oxygen detection, they generally contain large amounts of precious metal (ca. 20 wt%), which is not economically beneficial. Actually, high concentration of these complexes may induce the self‐quenching effect because the complexes are too close to each other, and MOFs doped with small amounts of precious metal complex are effective enough to detect oxygen. Lin et al. reported that phosphorescent Ir and Ru complexes decorated with two carboxylate groups can be doped into the framework of a remarkably stable MOF [Zr_6_O_4_(OH)_4_(bpdc)_6_] (UiO‐67) to achieve high oxygen sensitivity with much reduced use of precious metal (**Figure** **14**).^127^ UiO‐67 consists of 12‐connected Zr_6_O_4_(OH)_4_ clusters linking by bpdc^2−^ ligands into an **fcu** topology network. The M(ppy)*_x_*(22bpy)_3−_ *_x_* (M = Ir(III) or Ru(II), *x* = 1, 2, 3) derived linear dicarboxylate ligands were used to partially (0.48–2.98%) replace the bpdc^2−^ ligands in UiO‐67. The luminescence quenching experiments were conducted by pressing desolvated samples onto the surface of KCl pellets, then placing into quartz cuvettes equipped with vacuum/O_2_ port system. Upon exposure to 0.8 bar O_2_, the phosphorescence intensities of these MOFs were instantly quenched by 29–65%. The sensing efficiency of these MOFs with very small amounts of precious metal (0.3–0.9 wt%) are comparable with those of completely connected by precious metalloligands. The emission intensities were found to decrease with non‐linear responses to increasing oxygen pressure, which was attributed to the inhomogeneous distribution of phosphorescent sites in the solid samples.

**Figure 15:**
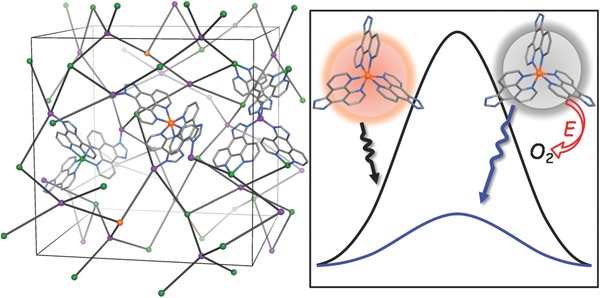
Simplified structure of *x*Ru:MAF‐34 (four‐coordinated and six‐coordinated Zn atoms are shown in violet and green, respectively, while the Ru atoms are highlighted in orange) and its luminescence quenching by O_2_ via energy transfer.

Sometimes, precious metal complexes can be directly used as bridging ligands, without sophisticated organic syntheses. [Ru(Hip)_3_]^2+^ is a classic phosphorescent complex used for constructing luminescent complexes for interacting with DNA. By using [Ru(Hip)_3_]Cl_2_ to replace part of the reactant Zn(OH)_2_, we synthesized a series of solid‐solution frameworks [Ru*_x_*Zn_7−_ *_x_*(ip)_12_](OH)_2_·guest (*x*Ru:MAF‐34, *x* = 0.10–0.16), in which Ru(II) partly replaced the octahedral Zn(II) (**Figure** **15**).^128^ Synthetic experiments using different Ru(II) doping ratios showed that unknown crystalline impurities appeared at *x* > 0.16, which can be attributed to the smaller radius of Ru(II) and the framework tension of MAF‐34. In other words, the higher Ru(II) doping ratios result in greater framework tension, finally leading to incompatibility between Ru(II) and the coordination network. Luminescence oxygen‐quenching study revealed that the sensing efficiencies of these materials increase along with the Ru(II) doping ratio, with a maximum of 88% quenching at 1 bar at *x* = 0.16, corresponding to *I*_0_/*I*_100_ = 8.3. This phenomenon is opposite with the common trends that the higher concentration of the luminophore results in poorer quenching efficiency due to self‐quenching. Luminescence lifetime measurements demonstrated that the higher Ru(II) doping ratios results in longer lifetimes. Probably induced by the increasing framework distortion with higher doping ratios, the coordination geometry of [Ru(ip)_3_]^−^ approaches more to the normal octahedral one, facilitating its phosphorescence emission. Sorption experiment showed relatively high O_2_ uptake of 0.20 mol L^−1^ at 1 bar and 298 K, which accounts for its relatively high oxygen‐sensing efficiency considering the relatively low precious metal contents (0.32–0.52 wt%) compared with full precious metalloligands. For comparison, the oxygen solubility in organic solvent C_29_HF_59_O_9_ and polymers ethyl cellulose are only 0.012 and 0.008 mol L^−1^, respectively.^129^, ^130^ By virtue of the suitable excitation and emission wavelengths of the Ru(II) complex, we fabricated a simple color‐changing ratiometric oxygen sensor by spraying 0.16Ru:MAF‐34 powder onto the outer surface of a blue light‐emitting diode (LED). In the absence of O_2_, 0.16Ru:MAF‐34 emits strong red light, which mixes with the leaked blue light to give a purple color. In the presence of O_2_, the red luminescence of 0.16Ru:MAF‐34 was quenched, and the device only emits blue light.

**Figure 16:**
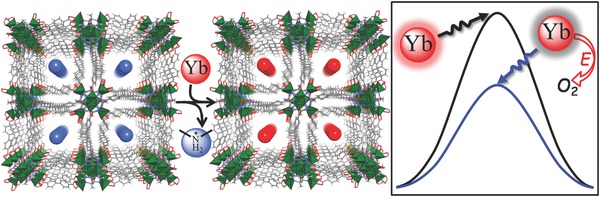
Post‐synthetic ion exchange from bio‐MOF‐1 to Yb^3+^@bio‐MOF‐1 and its luminescence quenching by O_2_ via energy transfer from Yb(III) to O_2_.

Besides Ru, Ir and other precious metals, lanthanide ions can be also used to construct phosphorescent oxygen‐sensing MOFs.^131^ For example, Rosi et al. reported that lanthanide ions can be encapsulated via postsynthetic exchange into a 3D anionic MOF (Me_2_NH_2_)_2_[Zn_8_O(ad)_4_(bpdc)_6_]·8DMF·11H_2_O (Ln^3+^@bio‐MOF‐1, Had = adenine). The near‐infrared (NIR) luminescence of the resultant material can be quenched by oxygen (**Figure** **16**).^132^ The bio‐MOF‐1 structure contains 1D channels of 7 × 7 and 10 × 10 Å^2^ with counter cations. Upon excitation with 340 nm UV light, Yb^3+^@bio‐MOF‐1 showed NIR emission maximum at 970 nm, which is benefit for sensitive detection in complex media such as biological samples since the background noise is very low. The luminescence of Yb^3+^@bio‐MOF‐1 at 970 nm was quenched by approximate 40% within 5 minutes upon exposure to O_2_ gas under ambient pressure, which was reversible and stable after several cycles of exposure to O_2_ and N_2_.

**Figure 17:**
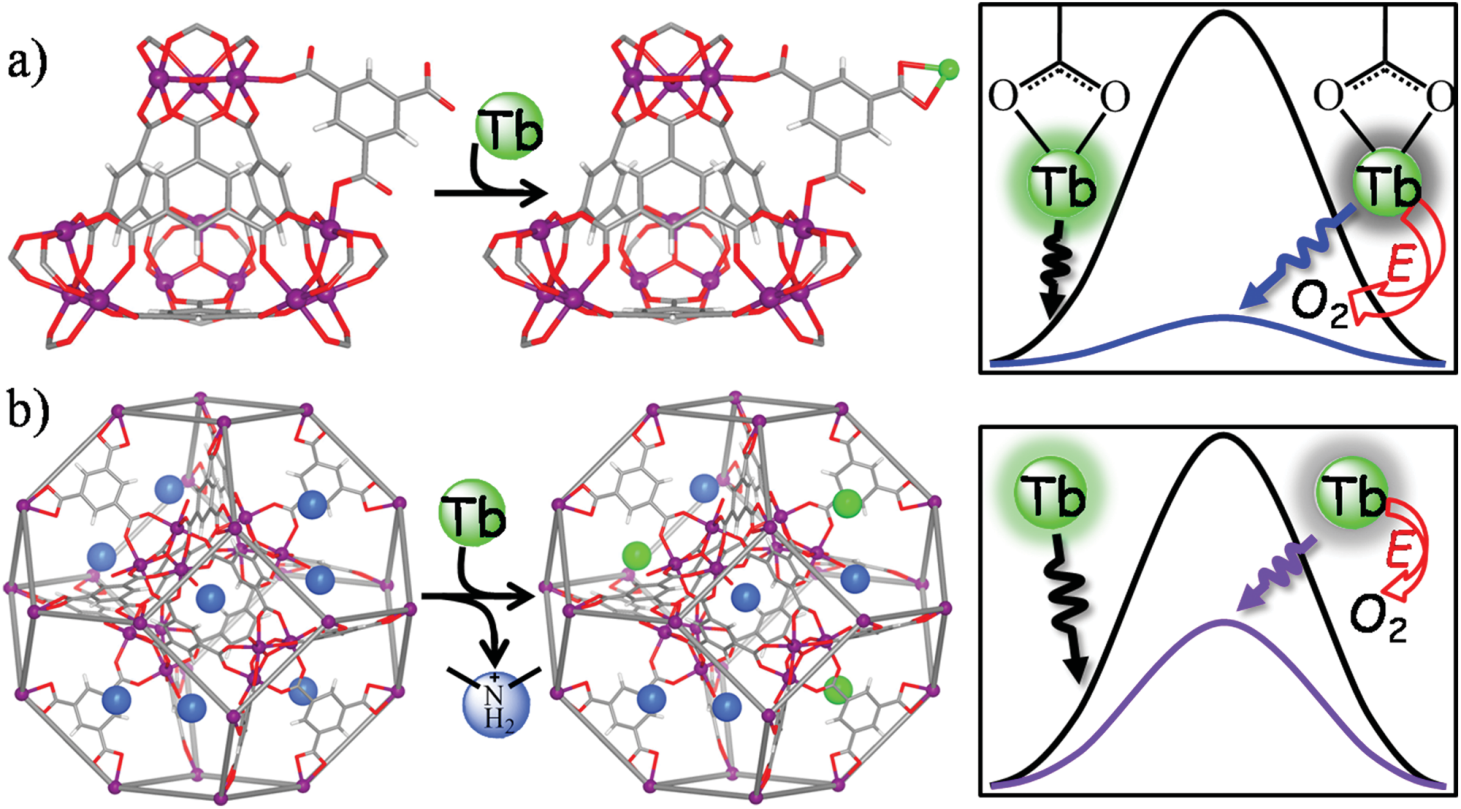
Comparison of the possible structures of a) Tb^3+^@MIL‐100(In) and b) Tb^3+^@CPM‐5, as well as their different luminescence quenching effects toward O_2_. The In and Tb atoms are shown in violet and bright green, respectively.

To increase the O_2_ sensitivity of lanthanide luminescence, sensitization of the lanthanide luminescence can be helpful. Qian et al. compared the oxygen sensing properties of two lanthanide doped MOFs.^133^ [In_3_O(OH)(H_2_O)_2_(btc)_2_] (MIL‐100(In), H_3_btc = 1,3,5‐benzenetri‐ carboxylic acid) is highly porous (void = 71%) with pore sizes of 20 and 26 Å. It was expected that there might be extra trimesic acid serving as the terminal molecule of In_3_O trimer, allowing the coordination of lanthanide ions to the terminal carboxylic acids via post‐synthesis method (**Figure** **17**). Immersing MIL‐100(In) into a DMF solution of Tb(NO_3_)_3_·6H_2_O gave Tb^3+^@MIL‐100(In), which showed characteristic Tb^3+^ emission peaks with a quantum yield of 16.8%. Upon exposure to variable O_2_ partial pressures in an N_2_ carrier gas, the luminescence of Tb^3+^@MIL‐100(In) was quenched in a linear manner with a quenching efficiency of 88% at 1 bar O_2_, corresponding to *I*_0_/*I*_100_ = 8.3. For comparison, [(CH_3_)_2_NH_2_][In_3_O(btc)_2_(H_2_O)_3_]_2_[In_3_(btc)_4_] (CPM‐5) possesses a moderately large solvent accessible void (48%) with small pore size of 4.89 Å, in which all carboxyl groups of the btc^3–^ ligand are deprotonated and (CH_3_)_2_NH_2_^+^ serves as the exchangeable charge‐balancing cation in pores. Because Tb(III) ions in Tb^3+^@CPM‐5 only serve as counter ions, it can be hardly sensitized by the coordination framework, giving a poor luminescence quantum yield of only 1.1%. Therefore, the luminescence of Tb^3+^@CPM‐5 film at 544 nm was only quenched by 47% at 1 bar O_2_, corresponding to *I*_0_/*I*_100_ = 1.89. While the luminescence quantum yields or lifetimes are critical for the different O_2_ sensitivities of the two MOFs, the difference of their pore sizes/volumes might also play a role.

Although phosphorescent MOFs containing precious metal or rare earth metal have made considerable progresses for oxygen sensing, and their metal contents can be reduced to very lower level, completely avoiding the use of precious metal is always highly demanded. On the other hand, organic fluorescent mole­cules with singlet excited state can also sense oxygen though their sensitivities are relatively low.^114^, ^134^, ^135^ Actually, theoretically speaking, fluorescence and phosphorescence have the same quenching probability when the luminophore is collided with oxygen. Their main difference for oxygen sensing could be the shorter lifetime of fluorescence. However, according to the Stern–Volmer (SV) equation, high O_2_ permeability of a MOF could compensate for the short luminescence lifetime.^120^

**Figure 18:**
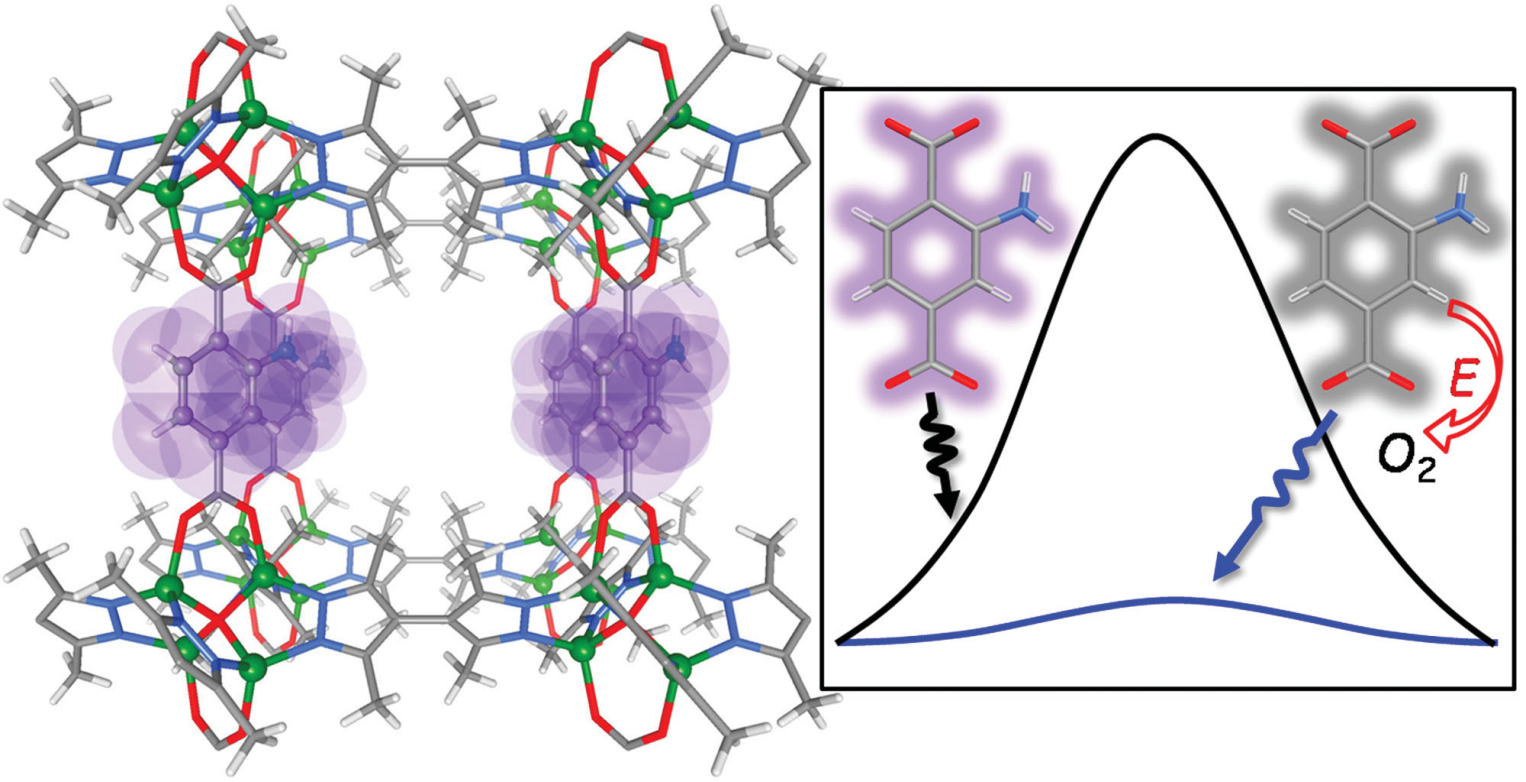
Structure of MAF‐X11 and its luminescence quenching by O_2_ via energy transfer from the abdc^2–^ ligand to O_2_.

We reported the first base‐metal MOF with singlet fluorescence for oxygen sensing, for which [Zn_4_O(bpz)_2_(abdc)] (MAF‐X11) was synthesized by simple reactants Zn(NO_3_)_2_, 2‐amino‐1,4‐benzenedicarboxylic acid (H_2_abdc), and 3,3′,5,5′‐tetramethyl‐4,4′‐bipyrazole (H_2_bpz).^136^ MAF‐X11 is a highly porous non‐interpenetrated **pcu** type coordination network composed of octahedral Zn_4_O(Rpz)_4_(RCOO)_2_ (Rpz^−^ and RCOO^−^ denote pyrazolate and carboxylate groups, respectively) clusters and two‐connected bpz^2−^ and abdc^2−^ linkers (**Figure** **18**). No visible photoluminescence can be observed for analogous MOFs, including [Zn_4_O(bpz)_2_(bdc)] (MAF‐X10),^137^ [Zn_4_O(bdc)_3_] (IRMOF‐1), and [Zn_4_O(abdc)_3_] (IRMOF‐3) because of their smaller π‐conjugation systems and/or close carboxylate‐carboxylate contacts leading to self‐quenching. MAF‐X11 exhibited intense fluorescent emission at ca. 470 nm upon excitation at 345 nm, which mainly originates from the abdc^2−^ linkers well separated by the bpz^2−^ linkers. Upon exposure to O_2_ at different pressures, the fluorescence of desolvated MAF‐X11 showed instant, reversible, and significant quenching. At 1 bar of O_2_, the fluorescence was quenched by 96.5% (*I*_0_/*I*_100_ = 28.5), which are significantly larger than for other oxygen sensitive phosphorescent MOFs and comparable with the highest values of hybrid materials composed of precious metal complexes. Sorption experiments showed relatively high O_2_ uptake of 0.21 mol L^−1^ at 1 bar and 298 K, as well as a large diffusion coefficient of 1.8 × 10^−5^ cm^2^ s^−1^, explaining its high oxygen‐sensing efficiency considering that its fluorescence lifetime (14.1 ns) is much shorter than for phosphorescent dyes. It was found that the fluorescence of IRMOF‐1, IRMOF‐3, and MAF‐X10 can be also quenched by O_2_, but their weak emission intensities and/or near ultraviolet emission colors make these O_2_ quenching properties unnoticeable.

**Figure 19:**
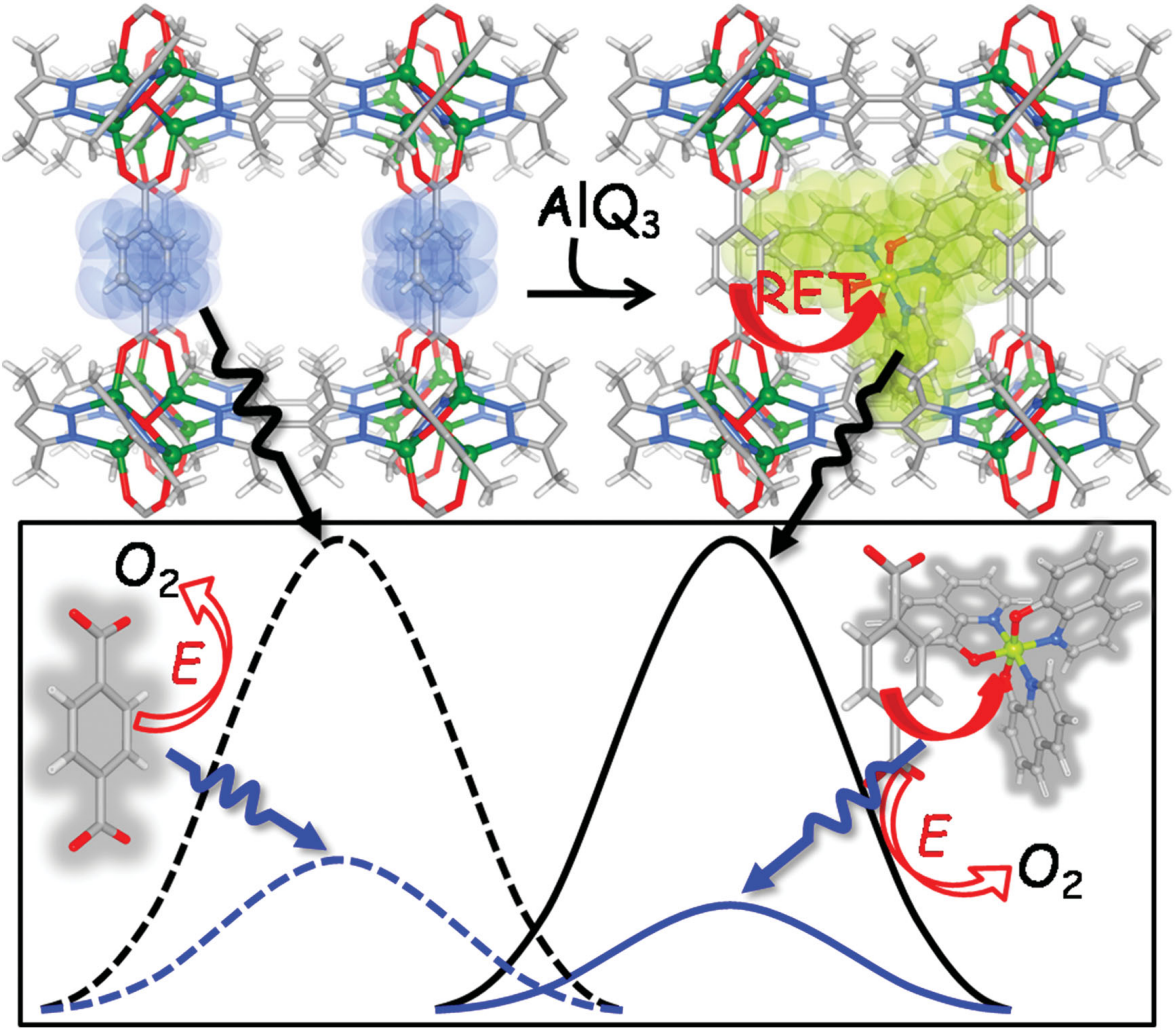
Comparison of the structures and luminescence and O_2_ quenching mechanisms of MAF‐X10 and AlQ_3_@MAF‐X10.

To utilize the oxygen sensitivity of these simple MOFs, we encapsulated a typical fluorescenct molecule tris(8‐hydroxyquinolinato)aluminium (AlQ_3_) into MAF‐X10 to obtain suitable excitation/emission wavelengths for O_2_ sensing (**Figure** **19**).^138^ The excitation maximum of AlQ_3_ (395 nm) overlaps the emission maximum of MAF‐X10 (400 nm), facilitating a resonance energy transfer process from MAF‐X10 to AlQ_3_. Under 345 nm excitaion, the host‐guest inclusion material AlQ_3_@MAF‐X10 showed yellow‐green fluorescence centered at 516 nm, being characteristic for AlQ_3_. The Stokes shift of 9606 cm^−1^ for AlQ_3_@MAF‐X10 is comparable with phosphorescent precious metal complexes. At 1 bar of O_2_, the fluorescence of AlQ_3_@MAF‐X10 was quenched by 88.5% (*I*_0_/*I*_100_ = 8.7), slightly higher than that of MAF‐X10 (84.5%, *I*_0_/*I*_100_ = 6.5), which was attributed to the significantly longer lifetime of AlQ_3_@MAF‐X10 (37.4 ns), since its oxygen permeability (4.4 × 10^−9^ mol cm^−1^ s^−1^ bar^−1^) is slightly lower than that of MAF‐X10 (5.0 × 10^−9^ mol cm^−1^ s^−1^ bar^−1^).

**Figure 20:**
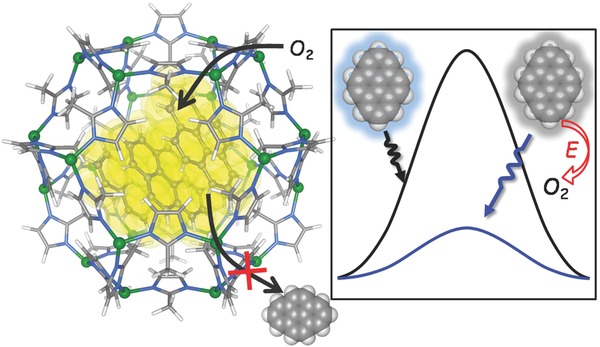
Structure of pyrene@MAF‐4, which allows luminescence quenching of pyrene via energy transfer to freely diffused O_2_, but forbids leakage/aggregation of pyrene because of the very small aperture of MAF‐4.

Pyrene is a good oxygen‐sensitive probe when dispersed in porous materials.^118^, ^139^, ^140^ However, this carcinogen and environmental pollutant tends to aggregate and/or evaporate at high temperature/low pressure.^141^ We encapsulated pyrene into the prototypi­cal metal‐organic zeolite SOD‐[Zn(mim)_2_] (Hmim = 2‐methylimidazole, MAF‐4 or ZIF‐8) via an in situ loading strategy, because MAF‐4 has large cavities with diameter of 11.4 Å and small apertures with diameter of 3.3 Å, which are ideal for immobilizing pyrene (3.4 × 7.2 × 11.6 Å^3^).^142^ Benefiting from the size match, the motion of pyrene can be well restricted while the diffusion of oxygen into MAF‐4 is allowed (**Figure** **20**). Indeed, thermogravimetry‐mass spectroscopy and gas chromatography‐mass spectroscopy analyses showed that no pyrene was released from MAF‐4 even at high temperature. Upon excitation at 344 nm, pyrene@MAF‐4 showed characteristic emissions of pyrene monomer and excimer, and the intensity ratio depends on the inclusion amount of pyrene. At 1 bar O_2_, the excimer emission intensities were quenched by 77.4–85.9% (*I*_0_/*I*_100_ = 4.42–7.09) for samples loaded with 1.8–0.047 pyrene molecules per cage, respectively. Moreover, since the small apertures of MAF‐4 prevent other large quencher molecules to pass through, the fluorescence of pyrene@MAF‐4 can not be interfered when exposed in the vapors of comment quenchers such as nitrobenzene, 2,6‐dinitrotoluene and 2,4‐dinitrotoluene. By virtue of the unique crystal growth behavior of MAF‐4, several methods can be used for fabrication of thin films of pyrene@MAF‐4 on the surfaces of various materials useful for sensing air flows.

**Figure 21:**
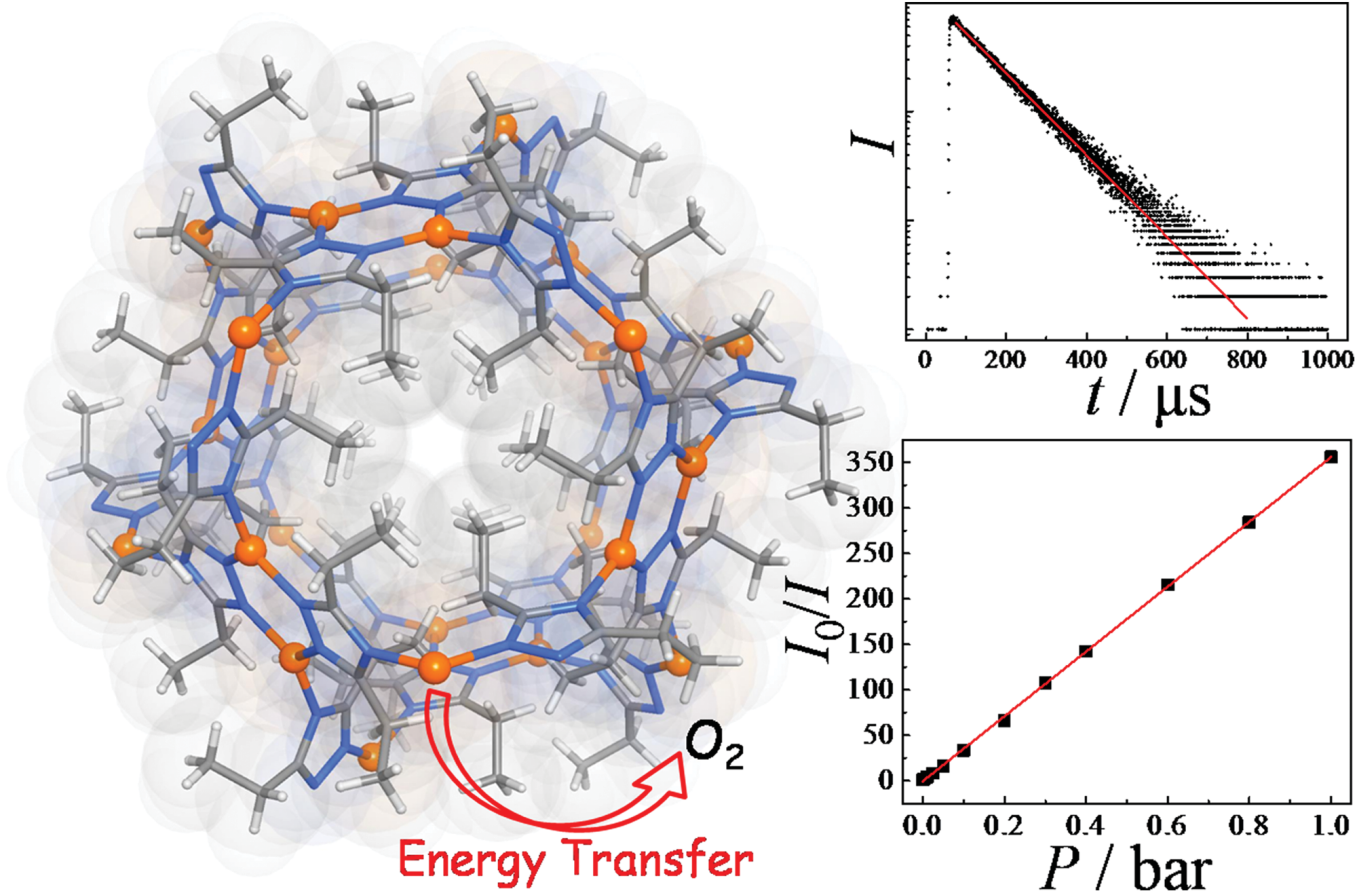
Structure of MAF‐2 and its time (luminescence lifetime) or O_2_‐pressure dependent luminescence.

Although some fluorescent materials have demonstrated high oxygen‐sensing efficiencies, phosphorescent dyes are still the most preferred choices for luminescence sensing, considering that phosphorescence based on triplet excited state shows high quantum yield and luminescent intensity, long lifetime and large Stokes shift, which can reduce the interference from scattered light and fluorescence background, meanwhile improve the sensitivity and signal to noise ratio.^47^, ^143^, ^144^ Many Cu(I) complexes are cheap phosphorescent materials, but they can be easily destroyed by air/moisture.^145^, ^146^ Based on the stable metal azolate framework system,^147^ we demonstrated that [Cu(detz)] (MAF‐2, Hdetz = 3,5‐diethyl‐1,2,4‐trizole), a Cu(I) triazolate framework with 3D intersecting micropores, can serve as an extremely oxygen‐sensitive material (**Figure** **21**).^148^ Upon excitation at 292 nm in vacuum, MAF‐2 showed broad and structureless emission maximum at 508 nm with Stokes shift of 14562 cm^−1^, which is larger than for most oxygen‐sensing materials (ca. 4000–10000 cm^−1^). The phosphorescence intensity of MAF‐2 were found to decrease linearly against the O_2_ pressure, with an extraordinarily high quenching of 99.72% at 1 bar (*I*_0_/*I*_100_ = 355.8). Besides the very long phosphorescence lifetime of 115.9(2) μs, the relatively large O_2_ solubility of 0.12 mol L^−1^ and diffusion coefficient of 1.4 × 10^−7^ cm^2^ s^−1^ were responsible for the high O_2_ sensitivity. Since MAF‐2 can be synthesized at room temperature by simply mixing the metal ion and the ligand, a counter‐diffusion crystal‐growth method was developed to grow microcystals on silicon rubber thin films. The soft membrane not only maintained original luminescence and oxygen‐sensing properties, but also further improved the chemical stability of MAF‐2.

Although O_2_ sensing based on luminescent MOFs have achieved numerous progresses, especially for improving/tuning the sensitivity and reducing the materials cost, these materials are still far from practical applications. Obviously, there are great needs and difficulties for luminescence sensing materials to enhance the O_2_ selectivity over other electron‐deficient and/or radical molecules. On the other hand, thin film and device fabrication are always great challenges for MOFs.

## 6 Conclusions

While MOFs have been extensively studied for gas storage, separation, and heterogeneous catalysis during the past decade, using luminescent MOFs for gas sensing is still developing and needs more attention. For practical applications, the ideal luminescent MOFs need to demonstrate high selectivity, high sensitivity, fast response, and full recyclability. Only a very small percentage of luminescent MOFs have been evaluated for gas sensing so far, especially for quantitative detection, considering the immense structural diversity of MOFs. To achieve high selectivity and sensitivity, the molecular sieving of MOFs, either by precise control over pore size or by framework flexibility, should be the key strategy. So far, the majority of luminescent MOFs have been focused on using bulk microcrystalline powders, but an increased attention is being paid on the fabrication of thin films and devices, because of their necessity for quantitative sensing of gas‐phase analytes. Based on the versatile structures and chemistry, as well as many viable types of sensing mechanisms such as the distance change between multiple luminophores, coordination to luminescent metal center, and electron/energy transfer between host and analyte, luminescent MOFs and related devices are very promising. And obviously, they are experiencing very fast growth for not only gas‐phase but also liquid‐phase chemical sensing.
